# *MYO5B* mutations in pheochromocytoma/paraganglioma promote cancer progression

**DOI:** 10.1371/journal.pgen.1008803

**Published:** 2020-06-08

**Authors:** Tajana Tešan Tomić, Josefin Olausson, Anna Rehammar, Lily Deland, Andreas Muth, Katarina Ejeskär, Staffan Nilsson, Erik Kristiansson, Ola Nilsson Wassén, Frida Abel

**Affiliations:** 1 Department of Pathology and Genetics, Institute of Biomedicine, University of Gothenburg, Gothenburg, Sweden; 2 Department of Mathematical Sciences, Chalmers University of Technology and Biostatistics, School of Public Health and Community Medicine, Institute of Medicine, Sahlgrenska Academy, University of Gothenburg, Gothenburg, Sweden; 3 Department of Surgery, Institute of Clinical Science, Sahlgrenska Academy, University of Gothenburg, Gothenburg, Sweden; 4 Region Västra Götaland, Sahlgrenska University Hospital, Department of Surgery, Section of endocrine and sarcoma surgery, Gothenborg, Sweden; 5 School of Health and Education, University of Skövde, Skövde, Sweden; 6 Sahlgrenska Cancer Center, Institute of Biomedicine, Sahlgrenska Academy, University of Gothenburg, Gothenburg, Sweden; Uniformed Services University, UNITED STATES

## Abstract

Identification of additional cancer-associated genes and secondary mutations driving the metastatic progression in pheochromocytoma and paraganglioma (PPGL) is important for subtyping, and may provide optimization of therapeutic regimens. We recently reported novel recurrent nonsynonymous mutations in the *MYO5B* gene in metastatic PPGL. Here, we explored the functional impact of these *MYO5B* mutations, and analyzed MYO5B expression in primary PPGL tumor cases in relation to mutation status. Immunohistochemistry and mRNA expression analysis in 30 PPGL tumors revealed an increased MYO5B expression in metastatic compared to non-metastatic cases. In addition, subcellular localization of MYO5B protein was altered from cytoplasmic to membranous in some metastatic tumors, and the strongest and most abnormal expression pattern was observed in a paraganglioma harboring a somatic *MYO5B*:p.G1611S mutation. In addition to five previously discovered *MYO5B* mutations, the present study of 30 PPGL (8 previous and 22 new samples) also revealed two, and hence recurrent, mutations in the gene paralog *MYO5A*. The three *MYO5B* missense mutations with the highest prediction scores (p.L587P, p.G1611S and p.R1641C) were selected and functionally validated using site directed mutagenesis and stable transfection into human neuroblastoma cells (SK-N-AS) and embryonic kidney cells (HEK293). In vitro analysis showed a significant increased proliferation rate in all three *MYO5B* mutated clones. The two somatically derived mutations, p.L587P and p.G1611S, were also found to increase the migration rate. Expression analysis of MYO5B mutants compared to wild type clones, demonstrated a significant enrichment of genes involved in migration, proliferation, cell adhesion, glucose metabolism, and cellular homeostasis. Our study validates the functional role of novel *MYO5B* mutations in proliferation and migration, and suggest the MYO5-pathway to be involved in the malignant progression in some PPGL tumors.

## Author summary

Up to 25% of pheochromocytoma/paraganglioma (PPGL) cases develop metastatic disease with poor outcome and few treatment options. The disease mechanism is not fully understood, and to date there are no reliable markers to predict malignancy. We have recently discovered novel missense mutations in the non-conventional myosin 5 gene (*MYO5B*), an endosomal transport protein, which we now show enhances progression and migration in PPGLs. *MYO5B* mutations were preferentially found in patients with metastatic disease and SDH deficiency (germline *SDHB*-mutations). Abolished SDH activity result in a metabolic switch to aerobic glycolysis requiring increased glucose consumption. Since the *MYO5B* mutations were found to drive progression through downstream up-regulation of glucose metabolism genes, *e*.*g*. glucagon, we hypothesize that these mutations may fuel the pseudohypoxic state by altering glucose uptake in cancer cells. Our result is the first to link the myosin 5 genes to PPGL tumorigenesis. Further, it shows that the tumor progression route in PPGL is complex, with contribution from several genetic factors. An increasing number of studies show dysregulation and importance of the MYO5-proteins in cancer, but little is still known about the precise role and mechanism of mutations, hence more research in this area is needed.

## Introduction

Pheochromocytomas (PCCs) and paragangliomas (PGLs), commonly denoted PPGLs, are rare neural tumors derived from chromaffin cells of the adrenal medulla and extra-adrenal paraganglia. PCCs and sympathetic PGLs secrete excess catecholamines with associated cardiovascular morbidity and mortality, and early diagnosis of these neoplasia is therefore vital. A great majority of PPGLs are sporadic and non-metastatic tumors and cured by surgery; however, up to 25% of cases develop metastatic disease (*i*.*e*. presence of PPGL in non-chromaffin organs) with poor outcome and few treatment options [1–4]. The survival rate at 5 years for patients with metastatic PPGLs is often less than 50% [5], and markers of metastatic disease are limited. PPGL have the highest degree of heritability in human neoplasms (around 30%), and over one-third of PPGLs are associated with inherited cancer susceptibility syndromes such as multiple endocrine neoplasia type 2 (MEN2), von Hippel–Lindau disease (VHL), neurofibromatosis type 1 (NF1), and hereditary paraganglioma (PGL1-PGL5). Inherited mutations have been identified in more than 14 genes, most commonly in *VHL*, *SDHB*, *SDHD*, *NF1*, and *RET* [6]. The most frequently mutated genes in PPGL belong to a wide range of functional classes, including kinase receptor and signaling (*RET, NF1, HRAS*, and *MAX*); energy metabolism (*SDHA, SDHB, SDHC, SDHD, SDHAF2, FH*); cellular response to hypoxia (*VHL*, and *EPAS1* (also known as *HIF2A*)); endosomal signaling (*TMEM127*), and chromatin remodeling (*ATRX*). Germline and somatic mutations in these 14 susceptibility genes account for more than 60% of PPGL cases [7,8]. Several additional genes have been recently reported [4,9–15], and the identification of more tumor-driving genes and mutations in PPGL could facilitate diagnosis, treatment decision, and may provide new therapeutic options for patients [16].

**Fig 1:**
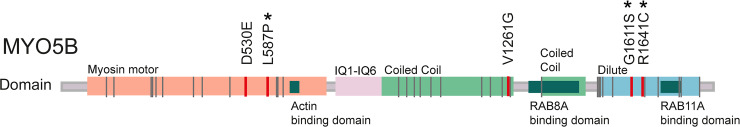
Localizations of mutations in *MY05B*. Schematic presentation of the *MYO5B* gene, located on chromosome 18, containing 40 exons encoding the MYO5B protein (NP_001073936, 1848 amino acids). MYO5B constitutes of a myosin head domain and a globular tail (dilute) domain. Protein domains and motifs are presented according to Qiu et al [76], and the RAB8A- and RAB11A-binding sites according to Roland et al [23]. Localization of the five missense mutations in MYO5B identified in Wilzén et al [14] are marked in red, and the three selected mutations (p.L587P, p.G1611S, and p.R1641C) analyzed in the current study are marked with a star. Previously identified *MYO5B* mutations in colon cancer are according to Letellier et al [32] and marked in grey. IQ = IQ motif, BD = binding domain.

Through large-scale sequencing of PPGLs, we recently reported recurrent mutations in the actin-dependent motor Myosin Vb gene; *MYO5B* [14] (Fig 1). Out of five novel nonsynonymous mutations identified, two somatically derived *MYO5B* variants (NP_001073936: p.L587P and p.G1611S) were found in metastatic sympathetic PGL cases from our data set [14]. Further screening of two public PPGL data sets [17,18] revealed three additional *MYO5B* mutations; one germline mutation in a metastatic PCC (p.R1641C), and two mutations in PCC tumor cases with metastatic potential (PASS score = 10) [19] or metastasized disease [18] (p.V1261G and p.D530E respectively). In addition, a novel somatic nonsynonymous mutation in isoform *MYO5A* (NP_000250.3: p.E926G) was discovered in a PCC tumor sample of our data set [14]. The non-conventional class V myosins, existing as three isoforms (Myo5A–C) in vertebrates, are motor proteins involved in intracellular cargo transport along actin-filaments. Class V myosins provide a molecular basis of a large number of essential cellular functions, such as cell motility, endocytosis, vesicle trafficking, and protein/RNA localization [20]. Myosin Vb (MYO5B) interacts with Rab-GTPases (Rab11, Rab8 and Rab11-FIP2), and plays an important role in vesicular transport and along the plasma membrane recycling pathway [21–23]. Loss-of-function mutations in *MYO5B* are common in microvillus inclusion disease (MVID) and cause disruption of cell polarity [24–27]. Emerging evidence show an important role of MYO5 proteins in several cancer types, where they often have an altered expression pattern [20,28]. For example, *MYO5A* expression is increased in a number of highly metastatic cancer cell lines and metastatic colorectal cancer tissues [29], and epigenetic downregulation of *MYO5B* has been reported to promote proliferation, invasion and migration in gastric cancer [30,31]. Additionally, *MYO5B* mutations and methylation-independent loss of *MYO5B* expression that matched disease progression was recently reported in colorectal cancer [32]. Mutations identified in PPGL cases are located in three domains of the MYO5B protein; the ATP dependent actin binding motor domain (p.D530E and p.L587P), coiled-coil rod domain mediating dimerization of motor protein and RAB8 binding (p.V1261G), and at the C-terminal globular tail domain that mediates cargo interactions and RAB11 binding (p.G1611S and p.R1641C) [33,34]. By functional prediction software, all five missense mutations were predicted to have an impact on the protein function, and none of the PPGL mutations were overlapping with previous reported *MYO5B* mutation spectra in colorectal cancer or MVID [14] (Fig 1). In this study we investigated the pathogenicity of the three most strongly predicted *MYO5B* mutations by functional *in vitro* studies. We also studied the protein and mRNA expression levels and subcellular location of MYO5B in primary PPGL tumors in relation to mutation status.

## Results

### Mutation status and MYO5B expression in primary PPGL tumors

**Fig 2:**
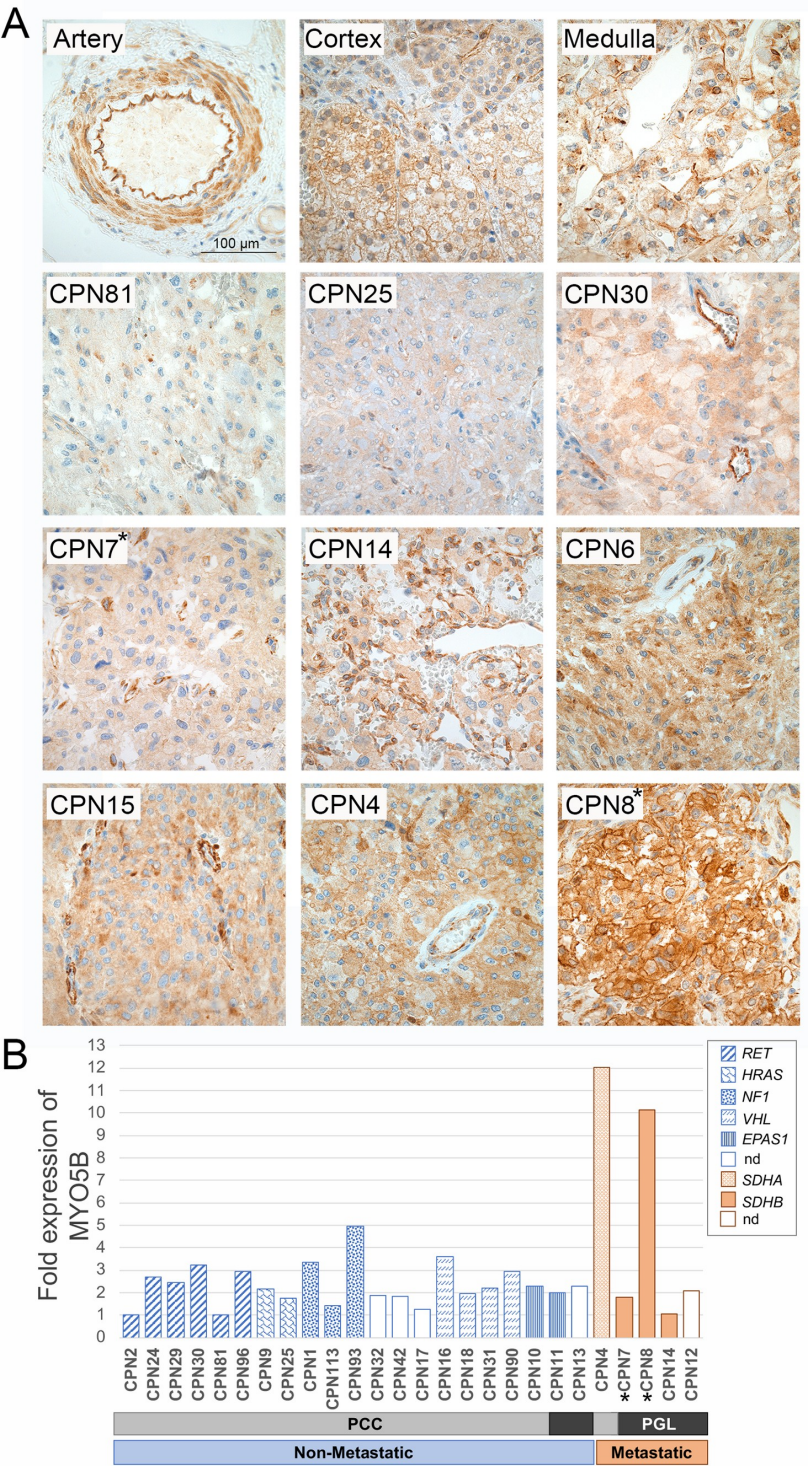
Expression of MYO5B in primary tumor tissue. Immunohistochemistry (A) using MYO5B antibody of normal adrenal gland (top row), 3 representative non-metastatic PCC tumors (second row) and 6 representative PGL metastatic tumors (two bottom rows). Two tumors harboring somatic *MYO5B* mutations were included; CPN7 (p.L587P) and CPN8 (p.G1611S). Pictures are taken at 40 x magnification using a Nikon ECLIPSE E1000M microscope and a ProgRes C7 camera, scale bar shown is 100μm. mRNA expression analysis (B) by microarray in 26 PPGL cases. The normalized relative expression values are presented as fold change compared to the non-metastatic case with lowest expression (CPN2), and each tumor sample is presented with its mutation status and diagnosis; PCC (light grey), PGL (dark grey), non-metastatic (blue), metastatic (orange). The two cases harboring *MYO5B* mutations are marked with a star. NA = not available.

MYO5B expression levels were assessed in primary PPGL tumors by immunohistochemistry (IHC; 23 cases) and mRNA expression microarray (26 cases). (Table 1). IHC of three normal adrenal glands displayed a strong membranous MYO5B staining in cortex, and strong cytoplasmic staining of endothelial cells, while adrenal medulla displayed a weak cytoplasmic MYO5B protein expression (Fig 2A). The staining intensity and % of labeled tumor cells was scored from 0–9 according to Klein et al. [35]. Weak or negative cytoplasmic MYO5B expression was observed in 6/14 non-metastatic tumors (score <4), while 6/14 showed intermediate expression (score 4–6) and 2/14 displayed a strong expression (score>6; Table 1; Fig 2A). In contrast, metastatic tumors displayed a significantly stronger expression; 3/9 cases with intermediate expression and 6/9 displaying strong MYO5B expression compared to non-metastatic tumors (p = 0.007 by Mann-Whitney, 2-sided). Furthermore, a deviant membranous location of the MYO5B protein was found in three metastatic cases (CPN3, CPN4, and CPN8). The strongest and most aberrant MYO5B expression pattern was observed in case CPN8 harboring the p.G1611S *MYO5B* mutation (Fig 2A). However, case CPN7, harboring the p.L587P mutation, demonstrated an intermediate cytoplasmic MYO5B staining similar to the majority of PPGL cases (Fig 2A). Tyrosine hydroxylase (TH) staining of adjacent tumor sections is provided in S1 Fig. The mRNA expression analysis of 26 cases (20 non-metastatic, 5 metastatic, and 1 multifocal tumor; Table 1) confirmed a higher expression in metastatic compared to non-metastatic tumors (2-fold, p = 0.260, t-test, 2-sided). This difference was mainly due to two metastatic cases (CPN4 and CPN8) showing a 10-fold higher MYO5B expression than the non-metastatic cases, and interestingly, these were the same two cases showing an aberrant membranous staining by IHC (Fig 2B). Unfortunately, the mRNA expression of the CPN3 case could not be elucidated due to no fresh frozen tissue available.

**Table 1:** Patient data, mutation status, and MYO5B expression.

Case ID	Sex	Diagnosis	Hereditary/ Sporadic	Syndrome	Metastatic/ Non-metastatic	Expr sub-type	Muta-tion status	MYO5B IHC			MYO5B mRNA Expr fold
								Expr Score	Localiza-tion	Expr Pattern	
CPN2	f	PCC	Hereditary	MEN2	Non-M	2	RET	6	Cytoplasm	Granular	1,00
CPN24	f	PCC	nd	NA	Non-M	2	RET	6	Cytoplasm	Granular	2,68
CPN29	f	PCC	nd	NA	Non-M	2	RET	3	Cytoplasm	Even	2,47
CPN30	m	PCC	nd	NA	Non-M	2	RET	6	Cytoplasm	Granular	3,24
CPN81	f	PCC	Syndromic	MEN2	Non-M	2	RET	3	Cytoplasm	Granular	1,03
CPN96	f	PCC	Syndromic	MEN2	Non-M	2	RET	3	Cytoplasm	Granular	2,93
CPN9	m	PCC	Sporadic	NA	Non-M	2	HRAS	2	Cytoplasm	Patchy	2,19
CPN25	f	PCC	nd	NA	Non-M	2	HRAS	6	Cytoplasm	Even	1,74
CPN1	m	PCC	Sporadic	NA	Non-M	2	NF1	6	Cytoplasm	Granular	3,36
CPN113	f	PCC	nd	NA	Non-M	2	NF1	nd	nd	nd	1,42
CPN93	m	PCC	Syndromic	NF1	Non-M	2	ni	nd	nd	nd	4,94
CPN32	f	PCC	nd	NA	Non-M	2	ni	0	Cytoplasm	Even	1,88
CPN42	f	PCC	nd	NA	Non-M	2	ni	nd	nd	nd	1,82
CPN17	m	PCC	nd	NA	MuPC	2	ni	6	Cytoplasm	Even	1,27
CPN16	f	PCC	nd	NA	Non-M	1	VHL	9	Cytoplasm	Granular	3,61
CPN18	m	PCC	Hereditary	VHL	Non-M	1	VHL	9	Cytoplasm	Even	1,94
CPN31	m	PCC	nd	NA	Non-M	1	VHL	3	Cytoplasm (neg)	Even	2,20
CPN90	m	PCC	nd	NA	Non-M	1	VHL	nd	nd	nd	2,94
CPN10	f	PCC	Sporadic	NA	Non-M	1	EPAS1	nd	nd	nd	2,31
CPN11	f	PGL	Sporadic, HPT	NE prod	Non-M	1	EPAS1	nd	nd	nd	1,99
CPN13	f	PGL	Sporadic	NA	Non-M	1	ni	nd	nd	nd	2,29
CPN4	m	PCC	nd	DA prod	M	2	SDHA	9	Membrane	Granular	12,04
CPN8	m	PGL	Hereditary	NA	M	1	SDHB	9	Membrane	Even	10,15
CPN3	f	PGL	Hereditary	NA	M	nd	SDHB	6	Membrane	Patchy	nd
CPN6	m	PGL	Hereditary	NA	M	nd	SDHB	9	Cytoplasm	Even	nd
CPN7	m	PGL	Hereditary	NA	M	1	SDHB	6	Cytoplasm	Even	1,79
CPN12	m	PGL	Sporadic	NA	M	1	ni	9	Cytoplasm	Even	2,09
CPN14	m	PGL	Hereditary	NF1	M	2	SDHB	9	Cytoplasm	Patchy & Granular	1,04
CPN15	f	PCC	nd	NA	M	nd	ni	9	Cytoplasm	Patchy	nd
CPN123	m	PGL	Hereditary	NA	M	nd	SDHB	6	Cytoplasm	Even	nd

Patient data of 30 primary tumors and expression data of MYO5B by Immunohistochemistry (IHC) and mRNA microarray analysis. m = male, f = female, PCC = pheochromocytoma, PGL = paraganglioma, Hereditary = Family history or confirmed germline mutation in one of 14 PPGL major susceptibility genes, Sporadic = confirmed somatic mutation in one of 14 PPGL major susceptibility genes, Syndromic = Associated syndromic features, no known heredity, HPT = Primary hyperparathyroidism. NF1 = Neurofibromatosis 1, MEN2 = multiple endocrine neoplasia type 2, VHL = Von Hippel Lindau syndrome, NE prod = overproduction of norepinephrine, DA prod = overproduction of dopamine by biochemical analysis of urine and/or plasma. M = Metastatic (presence of tumor metastases in non-chromaffin organs), Non = Non-metastatic (no presence of tumor metastases), MuPC = Multifocal pheochromocytoma. Expr subtype = expression subtype by unsupervised hierarchical clustering: 1 = pseudohypoxic, 2 = kinase signaling. MYO5B IHC Expr Score = MYO5B protein expression level in tumor cells based on semiquantitative scoring system by Klein et al.[35], ranging between 0–9 (0 = negative, <4 = weak, 4–6 = intermediate, >6 = strong staining). Location (membrane, cytoplasm, or cell nuclei) and staining pattern (even, granular, or patchy) is provided. MYO5B mRNA expression fold = microarray expression level presented as fold change relative to the case with the lowest expression (case CPN2). ni = not identified, nd = not determined.

Mutation analysis of 40 genes by exome sequencing, and complementary Multiplex Ligation-dependent Probe Amplification (MLPA) analysis of 6 genes, identified a causative PPGL mutation in 23 out of 30 cases; 6 *RET*-mutated, 3 *NF1*-mutated, 4 *VHL*-mutated, 2 *HRAS*-mutated, 6 *SDHx*-mutated (*SDHA*, *-B*, *-C*, and *-D*), and 2 *EPAS1*-mutated tumors (Table 1; S1 Table and S2 Table). In the 7 remaining cases no apparent explanatory mutations among the 14 major PPGL susceptibility genes could be found. Mutations in *RET*, *NF1*, *VHL*, and *HRAS* were solely found in patients with PCC tumors, while mutations in *SDH*-genes were mainly found in patients with PGL tumors or metastatic PCC (Table 1). The majority of metastatic cases (7/9) harbored *SDHB*-mutations, one case displayed a germline *SDHA*- promoter mutation, and in one metastatic case no apparent PPGL-associated mutation could be found. The MLPA-analysis of CPN12, 13, 32, 42 and 93 displayed a complete somatic *SDHB* deletion (exon 1–8) at allele frequencies between 0.2 and 0.5. This pattern could either be due to loss of the *SDHB*-region, or chromosome 1p-deletion which is a classical somatic event in PPGL. Also, in CPN93 presenting syndromic NF1, a deletion of the whole *NF1* gene was detected, This is most probably a secondary inactivation event occurring after a primary *NF1* mutation, whichever could not be detected in the current analysis. In sample CPN81, a *RET* mutation (p.C609Y) was found at an allele frequency (AF) of 0.98, indicating a loss of heterozygosity of the wild type allele in the tumor tissue. In addition to the established PPGL susceptibility genes, secondary mutations were found in the following 8 genes; *ARNT*, *DNAH17*, *MYCN*, *MYO5A*, *MYO5B*, *MYO9B*, *SLC25A11*, *VCL* (S2 Table). Somatic mutations in 8 cases (CPN1-9) have been previously reported [14], and in the current study including 22 additional cases the following new mutations were identified: *ARNT*:p.Q391K, *MYO5A*:p.P1194Q, *MYO9B*:p.H1726Y and *SLC25A11*:p.A200S. Hence, by this study we have also identified the *MYO5A* gene as recurrently mutated in PPGLs; *MYO5A*:p.E926G previously identified in case CPN1 (somatic, AF = 0.29, [14]) and *MYO5A*:p.P1194Q identified in case CPN29 (somatic, AF = 0.25; S2 Table). In addition, a third *MYO5A* missense mutation, NM_000259.3: c.5065G>A, p.V1689I was identified in case CPN15 (germline, AF = 0.51), but this variant was filtered out due to presence in normal population (AF = 0.23% in Genome Aggregation Database (gnomAD) ALL). All three *MYO5A* mutations were predicted to be damaging by at least 2 out of 3 functional prediction algorithms (i.e. Polyhen, SIFT, and/or MutationTaster).

Unsupervised hierarchical cluster analysis of 153 genes with highest variance over samples, subdivided the 26 PPGLs samples into the two classical expression subgroups; cluster 1 comprising the Pseudohypoxic tumors (*VHL*/*EPAS1*-, and *SDHx*-mutated), and cluster 2 comprising the kinase signaling tumors (*RET*-, *NF1*-, and *HRAS*-mutated; S2 Fig, S3 Table) [36]. The only exception was the CPN14 case harboring a *SDHB*-mutation but with clinical manifestation of NF1, which was assigned to cluster 2. In addition, the CPN4 case, harboring a *SDHA* promoter mutation, showed a unique expression profile which did not fit well into any of the major PPGL expression subgroups (S2 Fig). The *SDHA* promotor mutation (c.-7A>C, p.?; S2 Table) has been described once before [37], but its pathogenicity is still uncertain.

### Functional studies of stably transfected cell lines

**Fig 3:**
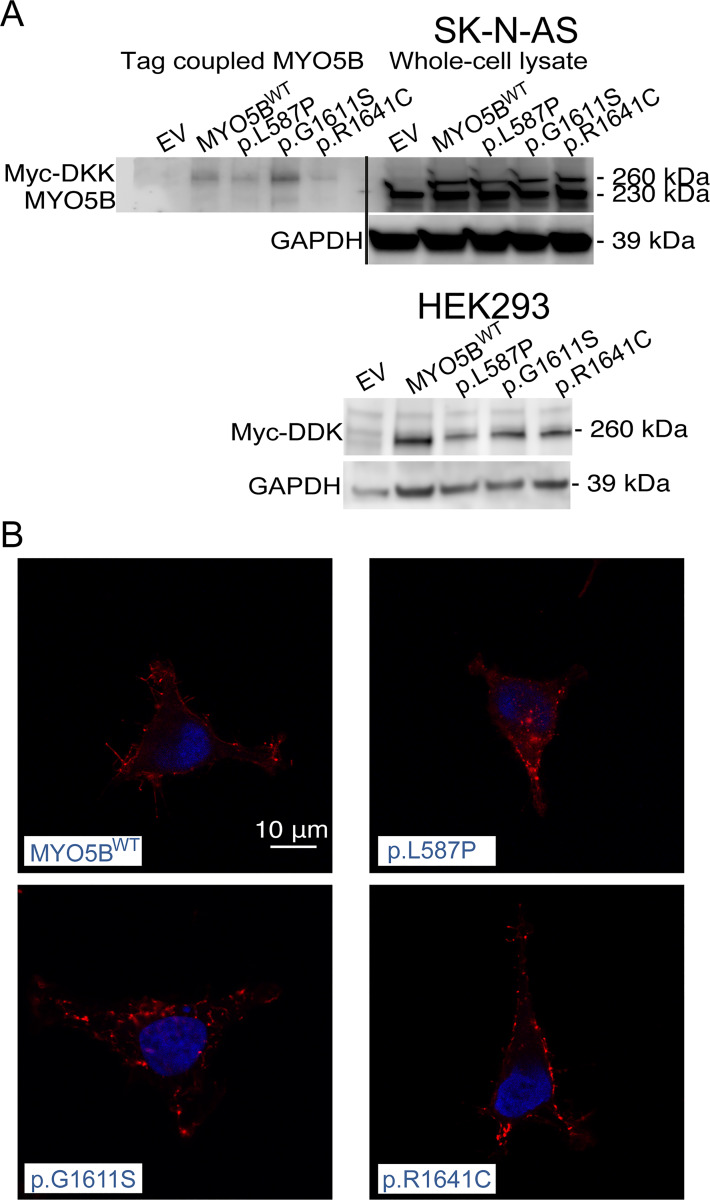
Expression and localization of MYO5B in cell lines. Western blot in SK-N-AS cells (A) showing MYO5B expression in whole cell protein lysate and FLAG M2-antibody coupled lysate (n = 1, top panel). Western Blot using FLAG M2 antibody in HEK293 (n = 1, bottom panel). GAPDH used as loading control. Localization of Myc-DKK (B) in HEK293 (63X, LSM700) in pCMV6-MYO5B_L587P-Myc-DDK (p.L587P), pCMV6-MYO5B_G1611S-Myc-DDK (p.G1611S), and pCMV6-MYO5B_R1646C-Myc-DDK (p.R1641C). Myc-DKK is depicted in red and DAPI staining of nuclei in blue. Scale bar shown is 10μm.

Three previously identified *MYO5B* missense mutations were selected based on theoretical prediction as damaging/deleterious/disease causing [14]; c.1760T>C, p.L597P; c.4831G>A, p.G1611S; c.4921C>T, p.R1641C (NM_001080467) (Fig 1). Stable clones of MYO5B mutants, MYO5B wildtype (WT) and empty vector constructs were generated in SK-N-AS and HEK293 cells. Western blot analysis of constructs showed a 230 kDa band representing endogenous expressed MYO5B protein, and 260 kDa band corresponding the additional size of the Myc-DKK-tag in transfected wildtype and mutated MYO5B proteins (Fig 3A). Co-immunoprecipitation of Myc-DKK-tagged proteins in SK-N-AS confirmed that 260 kDa band corresponded to the transfected MYO5B proteins (Fig 3A). By immunofluorescence the protein expression of mutated MYO5B (FLAG-tagged) showed a scattered cytoplasmic staining in small punctate spots, presumably localized to endosomal vesicles, in HEK293 cells (Fig 3B).

**Fig 4:**
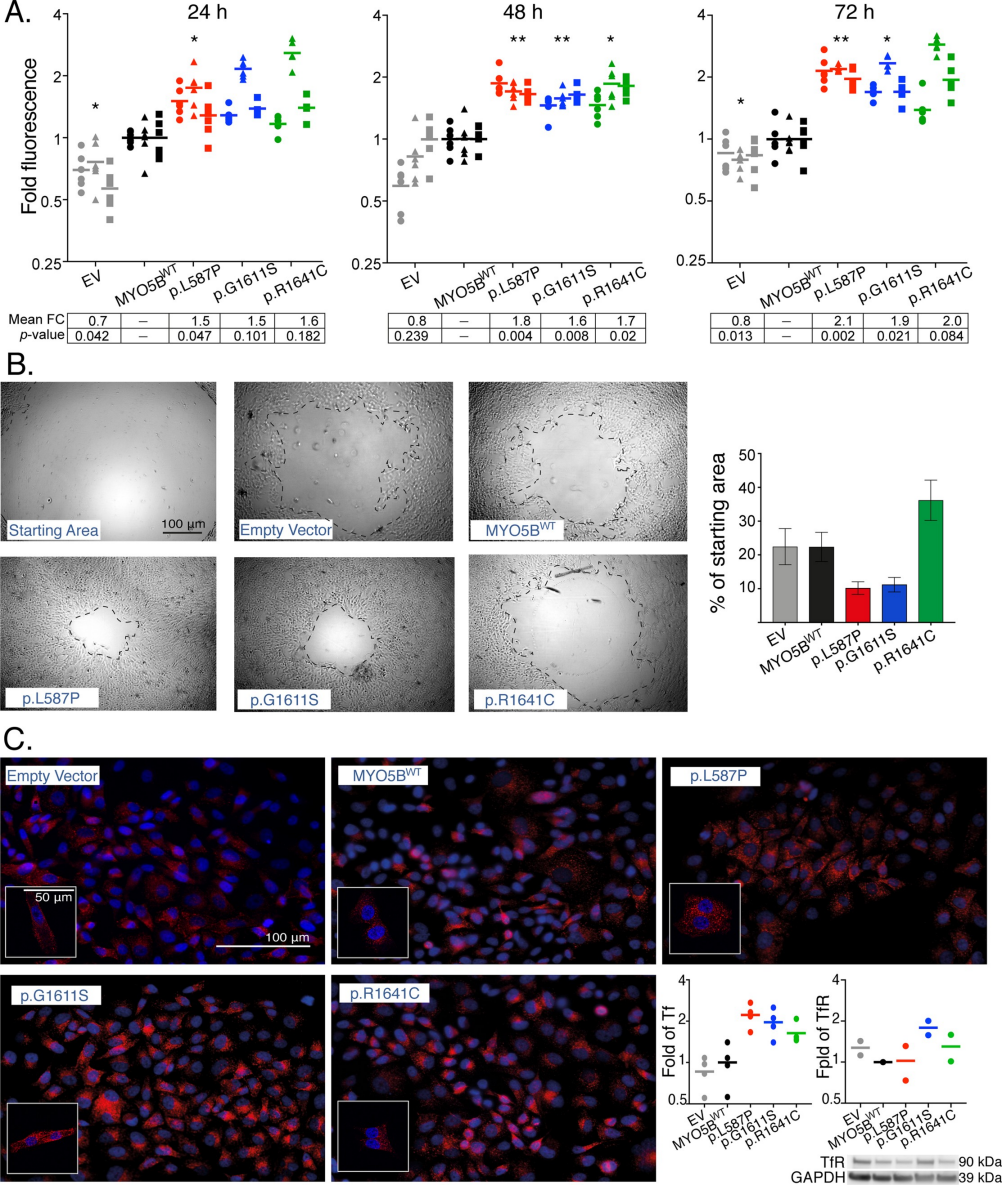
Proliferation, migration and endosomal recycling in *MYO5B*-mutants. Proliferation with CyQuant NF assay in SK-N-AS clones (A) shown as fold change at 24h, 48h, and 72h compared to mean MYO5B wild type (WT). Graphs show three independent experiments, each run in sextuplicates. Grey = empty vector, black = MYO5B^WT^, red = p.L587P, blue = p.G1611S, green = p.R1641C. Mean fold change (FC) and significance (*p*-value) compared to MYO5B^WT^ within each time point are presented (lower panel). * *p*<0.05, ** *p*<0.01, paired t-test. Migration (Oris) in HEK293 clones (B) shown as representable photos from light microscope (5X) of cell area remaining in MYO5B-mutants (p.L587P, p.G1611S, and p.R1641C), MYO5B^WT^, and empty vector at 24 h compared to the starting wound area. Scale bar shown is 100μm. Graph show percent wound area left after 24 h (mean±SEM, n = 2) in each construct. Transferrin uptake assay in SK-N-AS clones (C), with transferrin stained in red and nuclei stained in blue (DAPI). Pictures are taken with 40X Zeiss Axioscope 2 Plus fluorescence microscope, and small inserts show representative pictures taken with 63X, LSM700 confocal microscope. Scale bars shown are 50 μm and 100μm, respectively. Graphs plot fold change of corrected total cell fluorescence (CTCF) of transferrin (Tf) uptake (from four fields of view taken with 40X), and Western Blot analysis of the Transferrin receptor (TfR) expression (from two independent experiments) in mutants and empty vector compared to MYO5B^WT^ (n = 2). GAPDH was included as a loading control.

In order to assess the functional impact of the three *MYO5B* mutations in cultured, stably transfected cell lines, we investigated their ability to affect proliferation, migration and endosomal recycling. A significant increased proliferation (p<0.05) was found in all the three *MYO5B* mutations at 48h (p.L587P 1.8 fold; p.G1611S 1.6 fold; p.R1641C 1.7 fold), and the first and second mutations were also significant at 72h (p.L587P 2.1 fold; p.G1611S 1.9 fold) compared to MYO5B^WT^ and empty vector clones (Fig 4A). The scratch-wound assay showed an increased migration rate of p.L587P and p.G1611S mutants with only 10% of the wound starting area remaining after 24 h, compared to MYO5B^WT^ cells having 22% of the wound starting area remaining (Fig 4B). The p.R1641C mutation was not found to affect migration. The impact of mutations on endosomal recycling was explored by the transferrin trafficking assay. By visual inspection and fluorescence measurement of transferrin (Tf), the *MYO5B* mutations displayed a somewhat higher transferrin uptake (1.5–2 fold), most apparent in the cells harboring the p.L587P and p.G1611S mutations (Fig 4C). However, by relating the transferrin uptake to its receptor (TfR), the p.G1611S and p.R1641C mutants were found to express the transferrin receptor in almost the same proportion as transferrin, leaving p.L587P as the only mutation with slight impact on endosomal transport of transferrin (Fig 4C).

### Downstream expression analysis of *MYO5B* mutants

To elucidate the signaling pathways associated with *MYO5B* mutations in more detail, a transcriptome microarray analysis was performed in SK-N-AS constructs from three time points of proliferation (24h, 48h, and 72h) and from two replicated experiments. The global expression of each MYO5B mutant was compared to MYO5B^WT^ and empty vector constructs for each time point, respectively. First, we checked and confirmed an up-regulated expression of the *MYO5B* gene in the clones harboring either mutant or wild type *MYO5B* cDNA vector constructs compared to clones with empty vector (S4 Table). Next, the top-ranked differentially expressed genes from all three MYO5B mutants were filtered out, and analyzed for enrichment of cellular processes using the Gene Ontology tool GOrilla (http://cbl-gorilla.cs.technion.ac.il) [38] (S5 Table). The gene ontology analyzes showed significant (p<0.001) enrichment of terms involving migration, *i*.*e*. “positive regulation of migration”, “cell motility”, “cellular component movement” and “protein kinase C activity” (S6 Table). Moreover, Gene Set Enrichment Analysis (GSEA) [39,40] was performed on each MYO5B mutant’s merged gene list (ranked after mean fold change) analyzing 236 Hallmark and KEGG curated gene sets from the Molecular Signature database (http://software.broadinstitute.org/gsea/msigdb/index.jsp). The most enriched gene sets in the three *MYO5B* mutants were the following four up-regulated: “Hallmark Myc targets v1”, “KEGG Base excision repair”, “KEGG Basal transcription factors”, “Hallmark E2F targets”, and four down-regulated: “Hallmark TNFA Signaling via NFKB”, “Hallmark Inflammatory response”, “KEGG ECM Receptor interaction”, “KEGG Arrhythmogenic right ventricular cardiomyopathy ARVC” (S7 Table).

**Fig 5:**
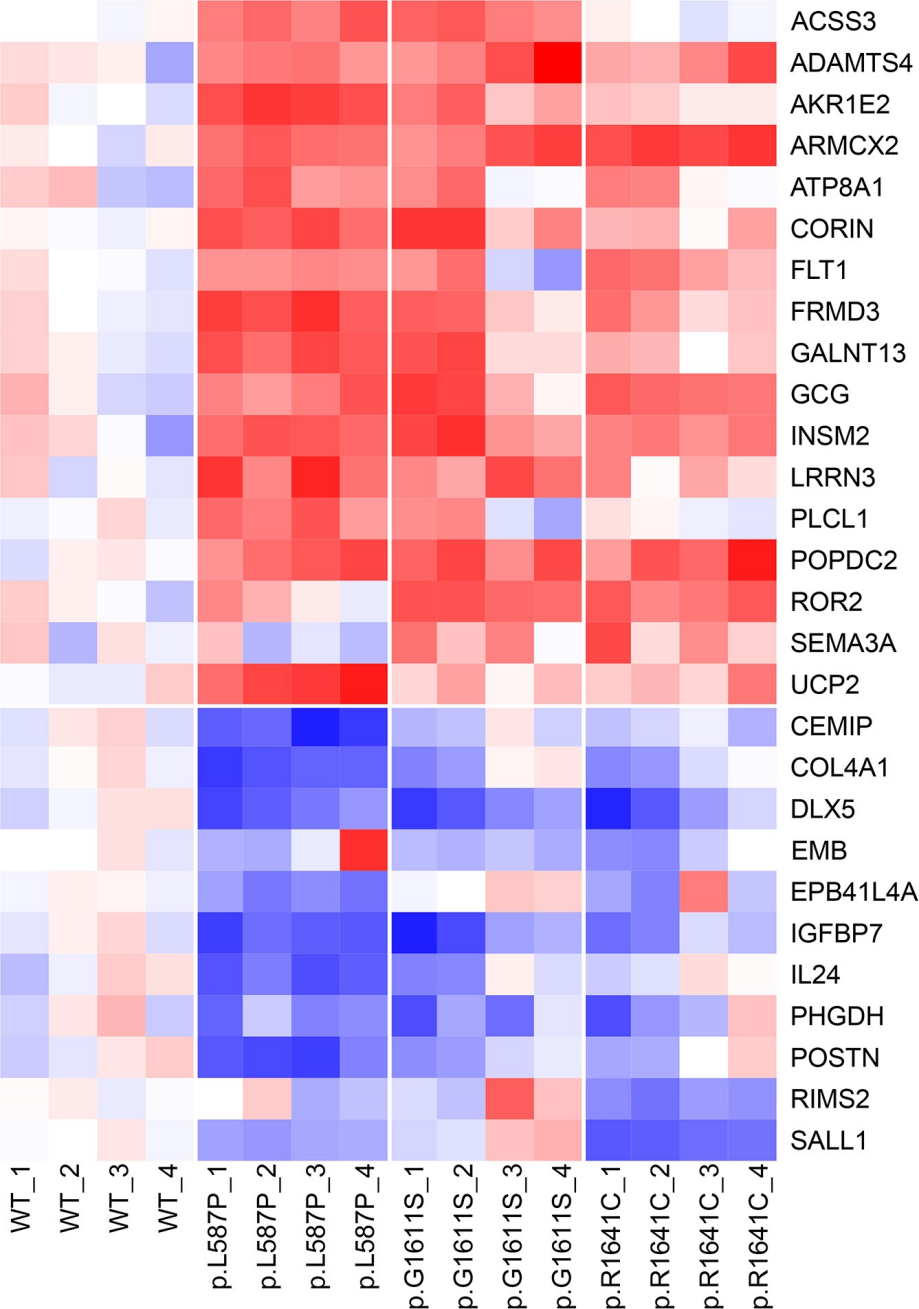
Heatmap of differentially expressed genes in MYO5B mutants. The heat map is based on qPCR data from 28 genes presented as dCt-values normalized gene-wise to a standard deviation equal to one, and the mean of the MYO5B^WT^ samples subtracted. Data include three MYO5B mutants (p.L587P, p.G1611S, p.R1641C) and MYO5B wild type (WT) run as four independent experiments (passages 1 = p21, 2 = p23, 3 = p28, 4 = p30 of SK-N-AS), analyzed at time point 48h in each proliferation experiment. Red = up-regulated, blue = down-regulated.

To verify the results, the 29 most differentially expressed genes (*i*.*e*. highest fold change) in all three mutants were selected from the top-ranked gene list; 17 up-regulated and 12 down-regulated genes compared to both MYO5B^WT^ and empty vector (Table 2). These genes were run by TaqMan quantitative real-time PCR (qPCR) for both the same two samples used in the microarray analysis (passages p23 and p30 of SK-N-AS cells) and in two additional experiments (passages p21 and p28), all from proliferation time point 48h (Table 2). Overall, the expression pattern could be verified in all four experiments for each mutant, except for one gene (*FILIP1L*) which was below detection limit by qPCR (Fig 5; Table 2). The highest fold change (ΔΔCt >1.5 in at least 3/4 passages) for all three MYO5B mutants was found in three up-regulated genes: *ARMCX2* (armadillo repeat containing X-linked 2), *GCG* (glucagon), *INSM2* (insulin transcriptional repressor 2); and in four down-regulated genes: *COL4A1* (Collagen Type IV Alpha 1 Chain), *DLX5* (distal-less homeobox 5), *IGFBP7* (insulin like growth factor binding protein 7), and *POSTN* (Periostin) (Fig 5; Table 2). Glucagon (*GCG*) and the insulin transcriptional repressor 2 (*INSM2*) were undoubtedly the two most up-regulated genes in all the three MYO5B mutants, with mean fold change of 7 and 14 respectively (Table 2).

**Table 2:** Differentially expressed genes in MYO5B-mutants.

Gene symbol	Micro array	qPCR verification		Gene name	Cellular function
		p23&p30	p21&p28		
GCG	2,45	3,09	2,63	glucagon	Glucose metabolism, homeostasis, and cell proliferation
INSM2	1,84	4,66	2,93	INSM transcriptional repressor 2	Glucose metabolism and homeostasis
GALNT13	1,51	1,62	1,28	polypeptide N-acetylgalactosaminyltransferase 13	Glycan biosynthesis and glycosylation
ARMCX2	1,33	1,88	1,92	armadillo repeat containing, X-linked 2	Mitochondria energy metabolism
FRMD3	1,07	1,19	1,09	FERM domain containing 3	Glucose metabolism and homeostasis
SEMA3A	1,03	0,24	0,27	semaphorin 3A	Neuronal and olfactory development
CORIN	0,99	1,57	1,43	corin, serine peptidase	Regulation of blood volume and pressure
FLT1	0,91	0,92	0,71	fms related tyrosine kinase 1	Angiogenesis and vasculogenesis, cell migration
FILIP1L	0,88	nd	nd	filamin A interacting protein 1 like	Focal adhesion, cytoskeleton, cell proliferation, and migration
ATP8A1	0,82	0,87	0,80	ATPase phospholipid transporting 8A1	Membrane transport, vesicle formation and trafficking, cardiac conduction, transport of glucose
ACSS3	0,81	1,12	1,14	acyl-CoA synthetase short-chain family member 3	Lipid synthesis and energy generation
UCP2	0,75	1,08	0,91	uncoupling protein 2	Mitochondria energy metabolism
AKR1E2	0,72	2,73	1,53	aldo-keto reductase family 1 member E2	Cell metabolism
PLCL1	0,62	0,47	0,49	phospholipase C like 1	Endocytosis process in neural cells
ROR2	0,61	1,65	1,29	receptor tyrosine kinase like orphan receptor 2	Cell proliferation, cell rigidity, bone formation
ADAMTS4	0,60	1,27	0,69	ADAM metallopeptidase with thrombospondin type 1 motif 4	Glycosylation, protein metabolism, cell migration
POPDC2	0,60	1,06	0,82	popeye domain containing 2	Heart rate dynamics
LRRN3	0,59	0,72	0,71	leucine rich repeat neuronal 3	Nervous system development
SALL1	-0,68	-1,09	-1,50	spalt like transcription factor 1	Transcriptional regulation and organogenesis
PHGDH	-0,69	-0,29	-1,08	phosphoglycerate dehydrogenase	Metabolism and electron transport activity
IL24	-0,73	-2,02	-1,68	interleukin 24	Cell prolieration
EPB41L4A	-0,76	-1,30	-0,43	erythrocyte membrane protein band 4.1 like 4A	Cytoskeleton and plasma membrane binding
EMB	-0,79	-0,10	-0,77	embigin	Glucose metabolism and cell proliferation
RIMS2	-0,90	-0,43	-0,19	regulating synaptic membrane exocytosis 2	Exocytosis and Rab3-binding
CEMIP	-1,35	-1,63	-1,65	cell migration inducing hyaluronan binding protein	Glycosaminoglycan metabolism, cell migration, endocytosis
COL4A1	-1,46	-1,63	-2,26	collagen type IV alpha 1 chain	Focal adhesion, ECM, cell proliferation, migration
POSTN	-1,47	-2,85	-2,83	periostin	Cell adhesion and migration, metastasis
IGFBP7	-1,90	-2,10	-2,65	insulin like growth factor binding protein 7	Glucose metabolism and cell adhesion
DLX5	-2,14	-3,18	-3,64	distal-less homeobox 5	Neural crest cell differentiation & cell proliferation

Microarray mRNA expression levels and quantitative PCR (qPCR) verification results of the 29 most differentially expressed genes in SK-N-AS stably transfected MYO5B-mutants. Microarray expression data is presented as log2 fold change of MYO5B mutants (MUT 1 = p.L587P, MUT 2 = p.G1611S, MUT 3 = p.R1641C) compared to MYO5B^WT^, calculated from a mean of all three mutants from 2 passages of SK-N-AS (p23 and p30) at three time points (24h, 48h, and 72h). The qPCR data is presented as ddCt (log2 normalized expression to three endogenous control genes) from a mean of all three mutants compared to MYO5B^WT^ at time point 48 hours of proliferation. Values from the same experiment as the micorarray (p23 and p30), as well as two additional experiments (p21 and p28), are presented.

Analyzing *GCG* or *INSM2* in primary tumors harboring *MYO5B* mutations (i.e. CPN7 and 8) or in tumors with increased *MYO5B* expression (i.e. CPN4 and 8) showed no specific up-regulation of these genes. However, a heat map of the *MYO5B* mutation associated gene transcripts revealed a differential expression between the two major subtypes of PPGLs (S3A Fig). Also, the expression of MYO5-associated RABs (*i*.*e*. *RAB8*, *RAB11*, *RAB11-FIP2*, *RAB25*) were found to differentiate the two PPGL clusters (S3B Fig).

## Discussion

Sequencing efforts and integrative genomic studies have started to uncover the genetic landscape of abnormalities that underlie PPGL tumors. However, functional verification work is needed to pinpoint the pathogenic disease-driving alterations in these tumors. We have previously published a study identifying novel recurrent *MYO5B* mutations in metastatic PPGL [14]. Here, we functionally verified the tumorigenic properties of three of these mutations by *in-vitro* studies in SK-N-AS and HEK293 cell lines. The human neuroblastoma cell line SK-N-AS was selected since it shares the same neural-crest origin, is wild type for all currently known PPGL susceptibility genes, and successfully used in several previous PPGL studies requiring a cell line of human origin [41–44]. Also, analyses in primary tumors revealed differential expression and altered sub-cellular localization of the MYO5B protein in metastatic PPGL, and mutation screening of additional PPGL cases identified recurrent mutations in the *MYO5A* paralog.

MYO5B is an actin-dependent intracellular vesicle transport myosin protein that has essential functions in the regulation of intracellular transport, membrane recycling, and cell movement. Mutations in *MYO5B* have been shown to disrupt cellular polarity in MVID [24,25]. Cell polarity is crucial for normal physiology and also plays a major role in development of tumor metastasis [45]. A link between the protein isoform MYO5A and cancer has already been established [29,46]. MYO5A show elevated expression in metastatic cancer cell lines derived from various tissue types, and is connected to tumor cell migration and metastasis *in vitro* and *in vivo* [29]. Emerging evidence also suggest that MYO5B proteins have an important role through differential expression in multiple cancer types [20,47]. Low protein levels of MYO5B has been shown to be associated with motility of gastric cells [30], and expression level and mutations in *MYO5B* has been reported as a powerful prognostic biomarker in colorectal cancer, which might help to stratifying patients for adjuvant therapy [30,32]. Here, we show a significantly stronger expression of MYO5B protein in metastatic tumors and much elevated mRNA expression levels (10-fold) in a subset of metastatic PPGL tumors. Moreover, an altered subcellular localization of the MYO5B protein to the membrane was observed in three metastatic cases, and the most prominent abnormal pattern was observed in the tumor case harboring the p.G1611S mutation. Although the exact mechanism of the altered protein localization is not known, we speculate that this could reflect a dysregulated exocytosis or endocytosis in these metastatic PPGLs. Nevertheless, the divergent pattern implies a role of MYO5B in the metastatic progression route.

The tumorigenic properties of the MYO5B mutations were demonstrated by a significantly increased proliferation rate for all three missense mutations (p.L587P, p.G1611S and p.R1641C). Furthermore, the p.L587P and p.G1611S mutations both showed increased migratory properties, and consistently the most significantly enriched GO-term among the differentially expressed genes in MYO5B-mutants was “positive regulation of migration”. With regard to the p.R1641C, it appears that this mutation had less effect on proliferation compared to the other two *MYO5B* mutations, and also did not increase migration. This is possibly due to the fact that p.R1641C was identified as a germline mutation in the primary case, while p.L587P and p.G1611S were originally discovered as somatic mutations [14, 17]. MYO5B is also implicated in regulating different pathways for endosomal recycling of proteins to the plasma membrane through interaction with Rab11, Rab8 and Rab11-FIP2 [21–23,48]. Although a moderately higher transferrin uptake in all three *MYO5B* mutants was observed, only p.L587P showed a slight effect on transferrin uptake after normalization to the transferrin receptor expression. Since the p.L587P is located in the actin binding myosin head, while p.G1611S and p.R1641C are located in the tail domain mediating Rab11 binding, the mechanisms may differ, which could also explain the different influence on transferrin receptor expression [23]. Taken together, the functional *in vitro* studies of MYO5B mutations suggests a gain of function or a dominant negative role of the mutants, enhancing mainly proliferation and migration.

The downstream transcriptome analysis of transfected SK-N-AS *MYO5B*-mutants showed a differential expression of genes involved in cell growth and proliferation, cell migration, cell adhesion, endosomal transport and glucose metabolism. Among the top-ranked down-regulated genes, *DLX5* (distal-less homeobox 5), has previously been found among the genes with the highest number of hypermethylated CpG sites in primary metastatic neuroblastoma tumors [49]. Also, *IGFBP7* (insulin-like growth factor binding protein 7), has been reported as epigenetically down-regulated and an independent prognostic factor in gastric cancer, leading to increased cell growth, invasion and migration [50]. Moreover, deletion of *IGFBP7* was found to increase proliferation in hepatocellular carcinoma by a constitutively active IGF signaling [51]. Interestingly the third most downregulated gene, *POSTN* (Periostin), is a multifunctional glycoprotein that plays a role in the adhesion process, in the migration of many cells, and importantly, in the epithelial-mesenchymal transition of cancer cells. Periostin has been linked to renal cell carcinoma, where it promotes migration and invasion via the integrin/focal adhesion kinase/c-Jun N-terminal kinase pathway [52]. Periostin has a prognostic value in multiple solid cancers, and has been suggested as a potential therapeutic target in human solid cancer [53]. Most conspicuous was the 14-fold increase in expression of *INSM2* (insulin transcriptional repressor 2) and the 7-fold increase in expression of *GCG* (glucagon) in all three MYO5B mutants. The pancreatic islets regulate glucose metabolism through secretion of islet hormones such as insulin and glucagon, and INSM2 is a direct target of Ngn3 and NeuroD1, two crucial transcriptional factors involved in human diabetes and pancreatic islet development [54]. Deletion of Insm2 in mice resulted in reduced insulin secretion and glucose intolerance [55]. *INSM2* is also called Insulinoma-associated gene 6 (*IA6*); Insulinomas are rare neuroendocrine tumors of the pancreas that are usually sporadic, but may occur in association with multiple endocrine neoplasia type 1 (MEN1) syndrome [56]). Also, glucagon signaling is shown to significantly stimulate proliferation in colon cancer cell lines [57]. Metformin, one of the most commonly used insulin sensitizers, has been demonstrated to have anti-proliferative effects in several human malignancies [58,59]. Furthermore, metformin has recently been shown to suppress proliferation in rat pheochromocytoma cell line PC12 [60]. Thus, the increased proliferation rate seen in the MYO5B mutants might be driven through altered energy metabolisms.

Interestingly, the glucagon receptor (GCGR) was found among the 153 classifier genes with the highest variance between PPGL subtypes, and has previously been reported as discriminative between SDHB- deficient tumors (high expression) and VHL- deficient tumors (low expression) [61], which is in line with our data. Altered metabolism is a key feature of the pseudohypoxic PPGLs [62] and has been linked to the Warburg effect with increased aerobic glycolysis [63]. The alternative energy-generation pathway is somewhat less efficient, requiring a much larger cellular influx of glucose to maintain the energy needs in tumor cells, and the increased glucose consumption can be exploited for diagnostic purpose [64]. Provocative tests using glucagon has previously been performed in patients with undiscovered PPGLs, and these tests have been reported to lead to multi-organ failure or hypertensive emergency in some cases [65–67]. This, together with the low sensitivity of the glucagon test, and diverse expression of the glucagon receptor in different PCC syndromes has led to recommendations to not use this test in clinical practice [68]. In light of our results, the possible direct involvement of glucagon in increasing proliferation and in fueling the glucose-dependent oncogenic state in specific subtypes of PPGLs, especially SDH-deficient tumors [69], might add to this list of arguments.

## Conclusion

An increasing number of studies show dysregulation of the MYO5-pathway in tumorigenesis and malignancy. In the present study we have functionally verified the tumorigenic role of three novel *MYO5B* mutations through their impact on proliferation and migration. The high expression and altered subcellular localization of MYO5B protein in a few malignant tumors speaks for an important role in progression of PPGL. Also, this study identified additional recurrent mutations in the *MYO5A* paralog, adding evidence of the MYO5-pathway's involvement in the tumorigenesis of PPGL. In conclusion, our study adds deeper insight into the complex genotype-phenotype correlation in PPGLs and also augments the emerging evidence of MYO5B's involvement in cancer.

## Materials and methods

### PPGL patients and primary tumor data

Fresh snap-frozen (SF) tumors (27 samples) and formalin-fixed paraffin-embedded (FFPE) tumor sections (3 samples) from 30 PPGL patients that underwent surgical resection between years 2000–2017 at the Department of Surgery, Sahlgrenska University Hospital, Gothenburg, Sweden, were included in the study (Table 1). Corresponding germline samples (blood or FFPE sections) were included for 15 of the patients. All tissue samples underwent routine pathological examination.

### Ethics Statement

The study was approved by the ethical review board in Gothenburg (Dnr: 239–13, Approved May 2013). All patients gave their written consent prior to surgery.

### Immunohistochemistry of primary PPGL

Paraffin embedded tumor sections and normal adrenal tissue (3–4 *μ*m) were deparaffinized, rehydrated and antigen retrieved in DAKO PT Link using EnVision FLEX Target Retrieval Solution (Dako). Immunohistochemistry (IHC) staining of tumor sections with antibody against MYO5B (1:250, Sigma) was performed in Dako Autostainer Link using EnVision FLEX with EnVision FLEX+ (LINKER) according to the manufacturer’s instructions (DakoCytomation). The stained sections were photographed using a Nikon ECLIPSE E1000M microscope with 40x objective and a ProgRes C7 camera. The scoring of MYO5B staining was evaluated independently by a board certified surgical pathologist (ONW) and a clinical geneticist (FA) using an Olympus BX51 light microscope. The MYO5B protein expression in tumor cells and adrenal tissue was scored based on the intensity of staining and % of stained cells according to the combinative semiquantitative scoring system provided by Klein et al.[35]. The scoring system range between 0–9 obtained from multiplication of the two following point scales: % cells (0% = 0, <33% = 1, 33–67% = 2, >67% = 3) and intensity (negative = 0, weak = 1; mild = 2, strong = 3). Moreover, the location (membrane, cytoplasm, or cell nuclei) and staining pattern (even, granular, or patchy) was inspected. The intensity of tumor sections was scored as strong when it had the same intensity as cortex and artery in the normal adrenal tissue and staining in more than 2/3 of the tumor cells (score 3*3 = 9). Immunostaining of Tyrosine hydroxylase (clone 1B5 from Leica Biosystems) in adjacent tumor sections was performed according to standard procedures in clinical routine to confirm the diagnosis of PPGL, and slides were scanned using a LEICA SCN400 scanner (S1 Fig).

### Mutation and expression analysis of primary PPGL

Genomic DNA from tumor tissue and blood was extracted according to Wilzén et al [14]. Mutation analysis of 40 target genes was performed by Illumina sequencing on 30 cases in either pair of tumor tissue (T) and normal tissue/blood (N) (15 cases) or as single tumor tissue (15 cases, S1 Table and S2 Table) [4,7,14,18,70–73]. Sequencing of cases CPN1-9 has previously been described [14]. The 22 new cases were sequenced by SureSelect v3/v5 (Agilent Technologies, CA) with paired-end (2*75-100bp) on a HiScanSQ (CPN10-15) or by SureSelect Clinical Research Exome v2 (CREv2; CPN16-CPN123), paired-end (2*100bp) on a NextSeq500 illumina sequencer. For details on variant calling and assessment see supplementary methods (S1 File). Only variants predicted to be damaging by functional prediction algorithms and/or previously reported in ClinVar (www.ncbi.nlm.nih.gov/clinvar/) or HGMD (portal.biobase-international.com) databases were included in the final mutation list (S2 Table). Sequence data has been deposited at the European Genome-phenome Archive (EGA), which is hosted by the EBI and the CRG, under accession number EGAS00001001601 (8 previous PPGL samples) and EGAS00001003991 (22 new PPGL samples). Further information about EGA can be found on https://ega-archive.org and "The European Genome-phenome Archive of human data consented for biomedical research"(http://www.nature.com/ng/journal/v47/n7/full/ng.3312.html). Nine cases with no apparent disease-associated pathogenic mutation by sequencing where further analyzed for exon/gene deletions or duplications by MLPA in 6 genes; *SDHA*, *SDHB*, *SDHC*, *SDHD*, *NF1*, and *VHL*. MLPA was performed on tumor DNA using the following four SALSA MLPA kits; P081-D1 & P082-C2 (NF1), P226-D1 (SDHx), and P016-C2 (VHL) (MRC-Holland www.mrcholland.com).

Expression analysis was performed on total-RNA from fresh tumor tissue by 44K Agilent Cy3/Cy5 2-color microarrays (see S1 File for details). Unsupervised hierarchical clustering of both samples and genes was performed using Omics Explorer 2.0 Beta from Qlucore (www.qlucore.se) using the Average linkage of Euclidian metric (each variable was normalized to mean = 0 and variance = 1). Using the filtering variance slider, transcripts with the lowest variance were filtered out until distinct subgroups appeared, resulting in a set of 153 genes (156 variables; S3 Table). Samples were divided into two cluster groups based on the dendrogram.

### MYO5B vector construct design

The full coding sequence of *MYO5B* wild type (NM_001080467) and *MYO5B* sequences harboring the three point mutations c.1760T>C (p.L597P), c.4831G>A (p.G1611S) and c.4921C>T (p.R1641C) were synthesized and cloned into the pCMV6 vector, and tagged with Myc-DKK tag by Invitrogen GeneART (Thermo Fisher Scientific). Five vector constructs; pCMV6-Myc-DDK (empty vector), pCMV6-MYO5B-Myc-DDK (MYO5B^WT^), pCMV6-MYO5B_L587P-Myc-DDK (p.L587P), pCMV6-MYO5B_G1611S-Myc-DDK (p.G1611S), and pCMV6-MYO5B_R1646C-Myc-DDK (p.R1641C) were sub-cloned and verified by DNA Sanger sequencing (GeneArt Gene Synthesis, Invitrogen, ThemoFisher Scientific).

### Cell culture and stable transfection

Human cancerous neuroblastoma cells (SK-N-AS, European Collection of Authenticated Cell Cultures, ECACC, SigmaAldrich) sharing the same embryologic neural crest origin as PPGL, and the non-cancerous embryonic kidney cells (HEK293, human embryonic kidney cell line, American Type Culture Collection, ATCC, USA), were cultured in high glucose DMEM (Thermo Fisher Scientific) supplemented with 10% fetal bovine serum (HEK293, Thermo Fisher Scientific) or 10% HyClone bovine growth serum (SK-N-AS, Thermo Fisher Scientific) in 37°C and 5% humidified CO_2_ using standard procedures. Prior to transfection, 4x 10^5^ cells were seeded onto 6-well plates one day ahead. Transfection of pCMV6-Myc-DDK vector constructs into SK-N-AS and HEK293 cells was performed using 2.5 μg of each construct, 7.5 μl Lipofectamine 3000, and 5 μl P3000 reagent (Thermo Fisher Scientific) following the manufacturer’s protocol. Five constructs per cell line were made; MYO5B^WT^, p.L587P, p.G1611S, p.R1641C, and empty vector. When cells reached confluency, they were seeded onto 10 cm plates and selection medium (500 μg/ml Geneticin, G418, Thermo Fisher Scientific), was added to the cells. Cells were sub-cultured with selection medium until experiments were conducted.

### Protein preparation and Western Blot

Transfected SK-N-AS and HEK293 were harvested and lysed using RIPA-buffer supplemented with phosphatase- and protease- inhibitors (Thermo Fisher Scientific). Protein lysates (30 μg) were resolved on 4–20% precast gels (Bio-Rad Laboratories) and transferred onto 0.45 μm PVDF membranes (Thermo Fisher Scientific). Western blot was performed using antibodies with ECL-detection (Supersignal West Maximum Fempto, Thermo Fisher Scientific) as follows: FLAG-DDK M2 mouse mAb (1:750, #F3165, Sigma Aldrich), GAPDH rabbit Ab (1:500, sc-25778, Santa Cruz Biotechnology), MYO5B Rabbit mAb (1:250, HPA040902, Atlas Antibodies), transferrin receptor (CD71) rabbit mAb (1:500, #13208, Cell Signaling Technology). Chemiluminescent signal from membranes were imaged using a LAS-400 imaging system (Fujifilm). The experiment was performed twice and quantified using Image Studio Lite v5.2.5 (https://www.licor.com/bio/image-studio-lite/). GAPDH was used as a loading control.

### Co-Immunoprecipitation

Proteins from SK-N-AS were co-immunoprecipitated using the Dynabeads co-immunoprecipitation kit (Thermo Fisher Scientific). FLAG-DKK M2 mouse antibody (1:100, Sigma Aldrich) was coupled to Dynabeads M-270 Epoxy beads. Antibody coupled beads (1.5 mg) and 1 mg protein lysate was used in the co-immunoprecipitation in order to form protein complexes. Co-immunoprecipitated proteins from SK-N-AS were then subjected to western blot as described above using MYO5B antibody in order to confirm FLAG-DKK/MYO5B association.

### Localization of FLAG-DKK

Transfected HEK293 cells were seeded at 2.5x 10^4^ cells/well onto MilliCell EZ slides (Merck Millipore) and allowed to attach during 48 h. Cells were fixed using 4% paraformaldehyde (HistoLab) and washed with Dulbecco’s PBS (+Mg^2+^/+Ca^2+^, Gibco, Life Technologies). Chambers were pretreated with AB-buffer containing 1% BSA, 0.5% Triton-X diluted in PBS during 5 minutes and stained with antibodies as follows: FLAG-DDK M2 mouse mAb (1:500), and Alexa Fluor 555 anti-mouse IgG (1:1000, A21424, Life Technologies) Chamber slide walls were detached and slides were mounted with Prolong Gold (Invitrogen, Life Technologies). Localization of proteins were visualized (63X) using LSM700 confocal microscope (Carl Zeiss).

### Cell proliferation

Transfected SK-N-AS was seeded at 5000 cells/well in sextuplicates in 96-well plates and incubated for 24h, 48h and 72h. Proliferation were measured using CyQuant Proliferation NF assay (Thermo Fisher Scientific), following manufacturers manual. Fluorescence was measured using Victor-3 multilabel reader (PerkinElmer), with excitation 485 nm and emission 530 nm. Three independent experiments were performed (SK-N-AS passage 22, 23 and 25, respectively).

### Cell migration

Transfected HEK293 cells were plated in quadruplicates at 4x10^4^ cells/well in Oris Cell Migration plates (Tebu-Bio) containing cell-seeding stopper. Cells were allowed to attach and reach 100% confluency during 48h and the cell stopper was removed. One well was photographed in a light microscope directly after removal of the cell stopper to get a starting area and all wells were photographed (5X) on Zeiss Axio Vert.A1 after 24h to determine the migration of the cells. The trial was repeated once, and only wells with cell layers showing an intact circular wound edge were included in the analysis (2–4 replicates/construct). Remaining area was measured with Image J v1.0 [74], and calculated as percent area left compared to well with starting area (100%).

### Transferrin assay

Transfected SK-N-AS cells were plated (2.5x10^4^) onto MilliCell EZ slides (Merck Millipore) and allowed to attach during 48 hours, then starved for 1,5 h in Life cell imaging solution (LCIS) (Thermo Fisher scientific). Cells were subsequently incubated for 15 minutes in 20 μg/ml Alexa fluor conjugated 555 human transferrin (T35352, Thermo Fisher) and thereafter fixed for 15 minutes in 4% paraformaldehyde solution (HistoLab). Chamber slide walls were detached and slides were mounted with Prolong Gold (Invitrogen, Life Technologies). Pictures were taken from 4–7 different areas of the slides with LSM700 confocal microscope (63X, Carl Zeiss) or Zeiss Axioscope 2 Plus fluorescence microscope equipped with Nikon DS- Qi1Mc camera (40X, Carl Zeiss, DAPI: 300 milliseconds (ms), Alexa fluor 555: 800 ms). For confocal settings see S1 File. Transferrin uptake was calculated in 4 areas by measuring total fluorescence staining from 40x pictures with Image J with Fiji package v1.0 [75], subtracted by the mean of background squares, and fluorescence was then divided by the number of cells in each picture.

### Microarray expression analysis of SK-N-AS mutant clones

Stably transfected SK-N-AS cells were seeded onto T75 flasks (1x10^6^ cells), then cultured and harvested at 24, 48 and 72 hours. The procedure was repeated twice (at passage 23 and passage 30). Cells were pelleted and RNA was extracted using Maxwell 16 LEV SimplyRNA kit (Promega) according to manufacturer’s protocol. RNA quality assessment was performed by measuring absorbance (A260/280; 1.9–2.1) on Nanodrop (Denovix DS-11 spectrophotometer) and RNA integrity number (RIN) values (>9.0) with High Sensitivity RNA Screen tape on 2200 Tape station (Agilent Technologies). Expression analysis was performed with Clariom S arrays for five constructs (empty vector, MYO5B^WT^, p.L587P, p.G1611S, and p.R1641C) at three time points of proliferation (24h, 48h, 72h) in duplicates by Eurofins Genomics (www.eurofinsgenomics.eu). Expression data on the 40 top-ranked differentially expressed genes from all three MYO5B mutants, *i*.*e*. genes present as differential expressed top-candidates in at least two out of three time points in at least two mutants, or at least in one time point in all three mutants, are presented in S5 Table. Next, Gene Ontology analysis by GOrilla (http://cbl-gorilla.cs.technion.ac.il [38]) and gene set enrichment analysis (GSEA, [39,40]) was performed on the gene lists. For further details on microarray and enrichment analyses see S1 File.

### Verification by quantitative real-time PCR (qPCR)

From the top-ranked genes in the microarray experiment, the 29 most differentially expressed genes in all three mutants were selected; absolute mean log2 fold change > 0.58 (corresponding to a 1.5 relative fold change) when compared to MYO5B^WT^. cDNA was synthesized using High-Capacity RNA-to-cDNA Kit (ThermoFisher Scientific) with 200 ng of extracted total RNA from the five stably transfected SK-N-AS constructs from four experiments each (p21, p23, p28, p30) at proliferation time point of 48h. Using custom-designed TaqMan Array Cards, qPCR analysis was performed for 32 genes (29 genes of interest, and 3 endogenous controls) in triplicates, run on a QuantStudio 12K Flex Real-Time PCR System according to manufacturer’s instruction (ThermoFisher Scientific). The three endogenous controls; *GAPDH*, *RPL37A*, *TBP*, were selected based on their low variance between samples from the expression microarray analysis. The design of the TaqMan Array Micro Fluidic Card is presented in S8 Table. The differential expression between MYO5B mutants versus MYO5B^WT^ constructs in each passage was calculated according to the ΔΔCt-method with normalization to the geometric mean of the three endogenous control genes.

### Statistical analysis

The protein expression of MYO5B by IHC and mRNA expression by microarray for metastatic versus non-metastatic tumors was analyzed using Mann-Whitney and t-test respectively (Table 1). Proliferation data points were tested for normality using Kolmogorv-Smirnov test (p>0.05 in all SK-N-AS clones and all time points), and proliferation was analyzed with paired t-test including the mean from each trial at 24, 48 and 72 hours. Western blot of TfR in SK-N-AS was analyzed with ANOVA, followed by Fisher’s LSD comparing MYO5-mutants to MYO5B^WT^ and empty vector. All statistical analyses were executed in IBM SPSS Statistics version 25.

## Supporting information

S1 Fig Tyrosine hydroxylase staining. Staining of Tyrosine hydroxylase (TH) in 23 patient tumor sections. Upper left panel shows a healthy adrenal gland (cortex and medulla) as a positive control. All tumor sections were positive for TH (21 cases), except for CPN3 and CPN12 which were positive for synaptophysin and chromogranin A. Pictures are scanned at 20X magnification using a Leica SCN400 scanner, scale bar shown is 50μm.
(TIF)

S2 Fig Unsupervised hierarchical clustering using a 153 discriminative gene set. Two major sample clusters appear; cluster 1with pseudohypoxic signaling (SDHB, VHL, and EPAS1 tumors) and cluster 2 with kinase signaling (RET, NF1 and HRAS tumors). The heat map color scale is based on standard deviations (sd) and ranges from +2 sd (red) to -2 sd (green). The status of malignancy and diagnosis are shown by grey and black squares; black = metastatic, dark grey = multifocal, light grey = non-metastatic; black = PGL, grey = PCC. Mutation status is marked as follows: red = *EPAS1*, orange = *SDHB*, yellow = *VHL*, blue = *RET*, turquoise = *NF1*, green = *HRAS*, brown = *SDHA*, white = not determined (nd). Two cases in cluster 2, one harboring a *SDHB*-mutation (CPN14) and one with no mutation identified (CPN93) showed NF1-manifestation.
(TIF)

S3 Fig Heat map of MYO5B-associated genes. Expression of 21 out of 29 most differentially expressed genes (A) and expression of 9 MYO5B-associated RABs (B) from the functional study of three MYO5B mutants. Tumor samples are displayed according to cluster subgroups. Genes are sorted by hierarchical clustering. The heat map color scale is based on standard deviations (sd) and ranges from +2 sd (red) to -2 sd (green). The status of malignancy and diagnosis are shown by grey and black squares; black = metastatic, dark grey = multifocal, light grey = non-metastatic; black = PGL, grey = PCC. Mutation status is marked as follows: red = *EPAS1*, orange = *SDHB*, yellow = *VHL*, blue = *RET*, turquoise = *NF1*, green = *HRAS*, brown = *SDHA*, white = not determined (nd).
(TIF)

S1 Table Gene-set for variant calling in PPGL. The 40 genes for calling of mutations in exomes were selected based on the following criteria; previously established or reported with germline and/or recurrent somatic mutations in PPGL (Buffet 2018 [9], Cascon 2015 [10], Dahia 2014 [7], Dwight 2017 [71], Fishbein 2017 [4], Papathomas 2014 [11], Remacha 2018 [12], Remacha 2019 [13], Yang 2015 [15], Yeh [77]), or recurrently occurring genes between lists of several PPGL studies (Castro-Vega 2015 [70], Flynn 2015 [72], Juhlin 2015 [73], Fishbein 2015 [18]), or recurrently mutated within our previous study (Wilzén 2016 [14]). Also, *MYO5B*-paralogs *MYO5A* and *MYO5C* were included. The 14 most commonly mutated genes in PPGL are marked with a star (*).
(PDF)

S2 Table Mutation analysis in 32 PPGL-associated genes. Mutation analysis by Exome sequencing and MLPA (see material and methods for details). Paired tumor tissue and normal samples (T & N) or single tumor samples (T) run by different library preparation kits (SureSelect v3 or v5 or Clinical research exome (CRE v2)). Variant filtering was performed by Alissa Interpret (Agilent Technologies) and somatic filtering in paired samples (T-N) was according to Wilzen et al., 2016 [14]. Major variant: pathogenic or likely pathogenic mutation in any of the 14 PPGL susceptibility genes and their allele frequency (AF) in normal and/or tumor sample. Present in Database: Variants previously reported in ClinVar (www.ncbi.nlm.nih.gov/clinvar/) or HGMD (portal.biobase-international.com) databases. Variants were defined as germline if occurring in the normal blood/tissue sample, and as somatic if only occurring in the tumor tissue sample. Other variants: secondary variants present in the 40-gene set occurring in AF>0.2, predicted to be damaging by at least 2 out of 3 functional prediction software (Polyhen, SIFT, and MutationTaster), and present <0.1% (germline) or 0% (somatic) in normal population databases. *Variants previously reported in COSMIC were included at lower AF. nd = not determined.
(PDF)

S3 Table Gene-set for expression clustering of PPGL tumors. The 153 genes with highest variance in 26 tumors samples, discriminating two expression clusters of PPGL tumors.
(PDF)

S4 Table MYO5B microarray mRNA expression of three MYO5B mutants versus empty vector. Microarray expression analysis of SK-N-AS constructs; MUT 1(p.L587P), MUT 2 (p.G1611S), and MUT 3 (p.R1641C) and wild type (WT) MYO5B compared to empty vector (EV). Calculations are based on measures from two SK-N-AS passages (p23 and p30) for each mutation and time point of proliferation (24h, 48h and 72h). t-statistic (t), significance (P.Value, and adj.p) and fold change (FC) from MUTvsEV group comparison.
(PDF)

S5 Table Top-ranked differentially expressed genes from microarray expression analysis of three MYO5B mutants. Average expression values (log2-transformed) from microarray mRNA differential expression analysis of three MYO5B mutants in SK-N-AS cells; MUT 1(p.L587P), MUT 2 (p.G1611S), and MUT 3 (p.R1641C) compared to wild type (WT) MYO5B. Calculations are based on measures from two trials and three time points of proliferation (SK-N-AS passages p23 and p30 for time point 48h and 72h, and one replicated SK-N-AS p23 trial at 24h). t-statistic (t), significance (P.Value, and adj.p) and fold change (FC) from the MUTvsWT group comparisons are presented per mutation and time point. Only the top -ranked genes, *i*.*e*. present in at least 2 time points for at least 2 mutants, or at least 1 time point for 3 mutants, are listed in the table (see S1 File for details).
(XLSX)

S6 Table Gene ontology enrichment analysis on top differentially expressed genes. The top-ranked differentially expressed genes (see S5 Table) from all three MYO5B mutants were analyzed using GOrilla (Gene Ontology enRIchment anaLysis and visuaLizAtion tool, http://cbl-gorilla.cs.technion.ac.il) with all genes present on the microarray used as background. N = total no. of genes recognized by Gorilla and associated with GO terms, B = no. of genes associated with the specified GO term, n = no. of genes recognized by GOrilla in the target set (one gene annotation including several genes and one gene not associated with any GO terms were excluded), b = no. of genes in the target set associated with the specified GO term. P-value threshold < 10^-3.
(PDF)

S7 Table Gene set enrichment analysis (GSEA) of ranked gene lists. Gene Set Enrichment Analysis (GSEA) performed on expression gene lists (ranked after mean fold change) for each mutant; MUT1 (p.L587P), MUT2 (p.G1611S), and MUT3 (p.R1641C), using 236 gene sets (50 H Hallmark, and 186 C2: KEGG curated gene sets) from the Molecular Signature database (http://software.broadinstitute.org/gsea/msigdb/index.jsp). The overlapping gene sets among the 20 top-ranked with up-regulated genes (UP) and 20 top-ranked with down-regulated genes (DOWN) for each mutations are listed.
(PDF)

S8 Table TaqMan Array Micro Fluidic Cards design. Assay-ID and target information for TaqMan primers and probes used for qPCR verification of the 29 most differentially expressed genes in MYO5B mutants. INV = Inventoried assay, MTO = Made-to-order assay.
(PDF)

S1 File Supplementary methods on variant calling, microarray analyses, and confocal microscope settings. (PDF)
