# Supplementary figures and images for: MYO5B mutations in pheochromocytoma/paraganglioma promote cancer progression
Source: PLoS Genet. 2020 Jun 8;16(6):e1008803. doi: 10.1371/journal.pgen.1008803 (PMC7329139; doi:10.1371/journal.pgen.1008803)

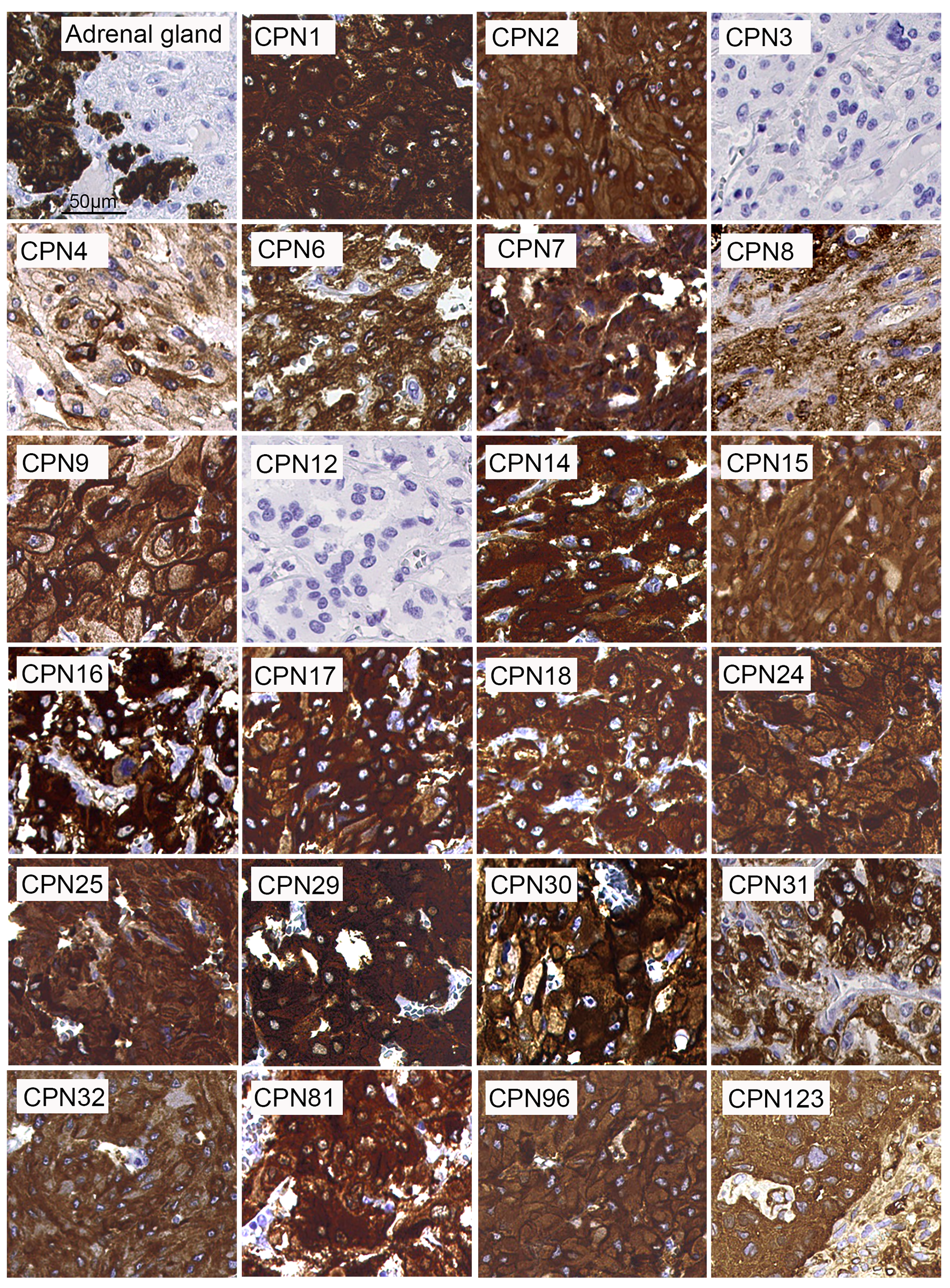

Supplement: S1 Fig — Staining of Tyrosine hydroxylase (TH) in 23 patient tumor sections. Upper left panel shows a healthy adrenal gland (cortex and medulla) as a positive control. All tumor sections were positive for TH (21 cases), except for CPN3 and CPN12 which were positive for synaptophysin and chromogranin A. Pictures are scanned at 20X magnification using a Leica SCN400 scanner, scale bar shown is 50μm. (TIF) [file pgen.1008803.s001.tif]

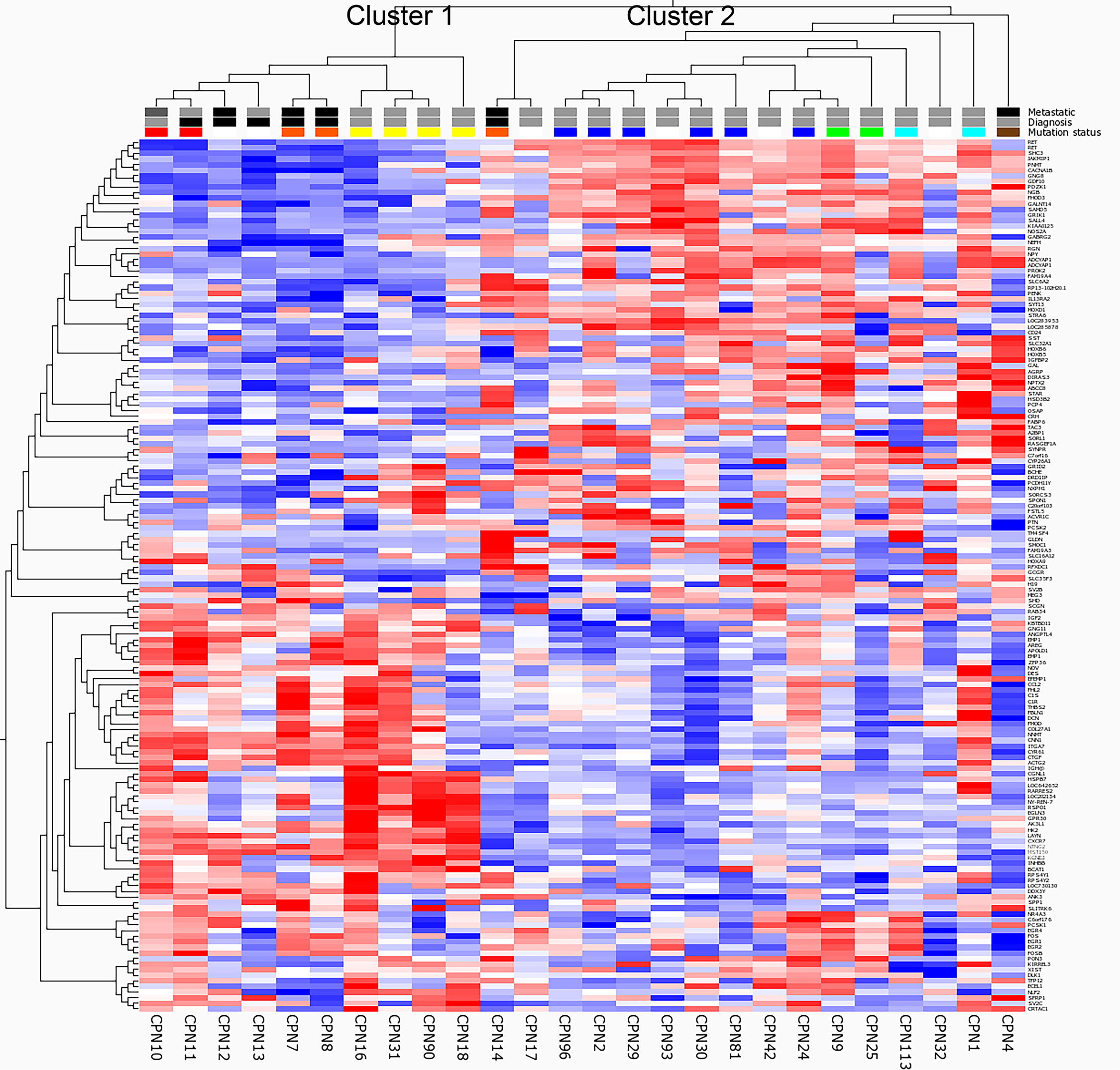

Supplement: S2 Fig — Two major sample clusters appear; cluster 1with pseudohypoxic signaling (SDHB, VHL, and EPAS1 tumors) and cluster 2 with kinase signaling (RET, NF1 and HRAS tumors). The heat map color scale is based on standard deviations (sd) and ranges from +2 sd (red) to -2 sd (green). The status of malignancy and diagnosis are shown by grey and black squares; black = metastatic, dark grey = multifocal, light grey = non-metastatic; black = PGL, grey = PCC. Mutation status is marked as follows: red = EPAS1, orange = SDHB, yellow = VHL, blue = RET, turquoise = NF1, green = HRAS, brown = SDHA, white = not determined (nd). Two cases in cluster 2, one harboring a SDHB-mutation (CPN14) and one with no mutation identified (CPN93) showed NF1-manifestation. (TIF) [file pgen.1008803.s002.tif]

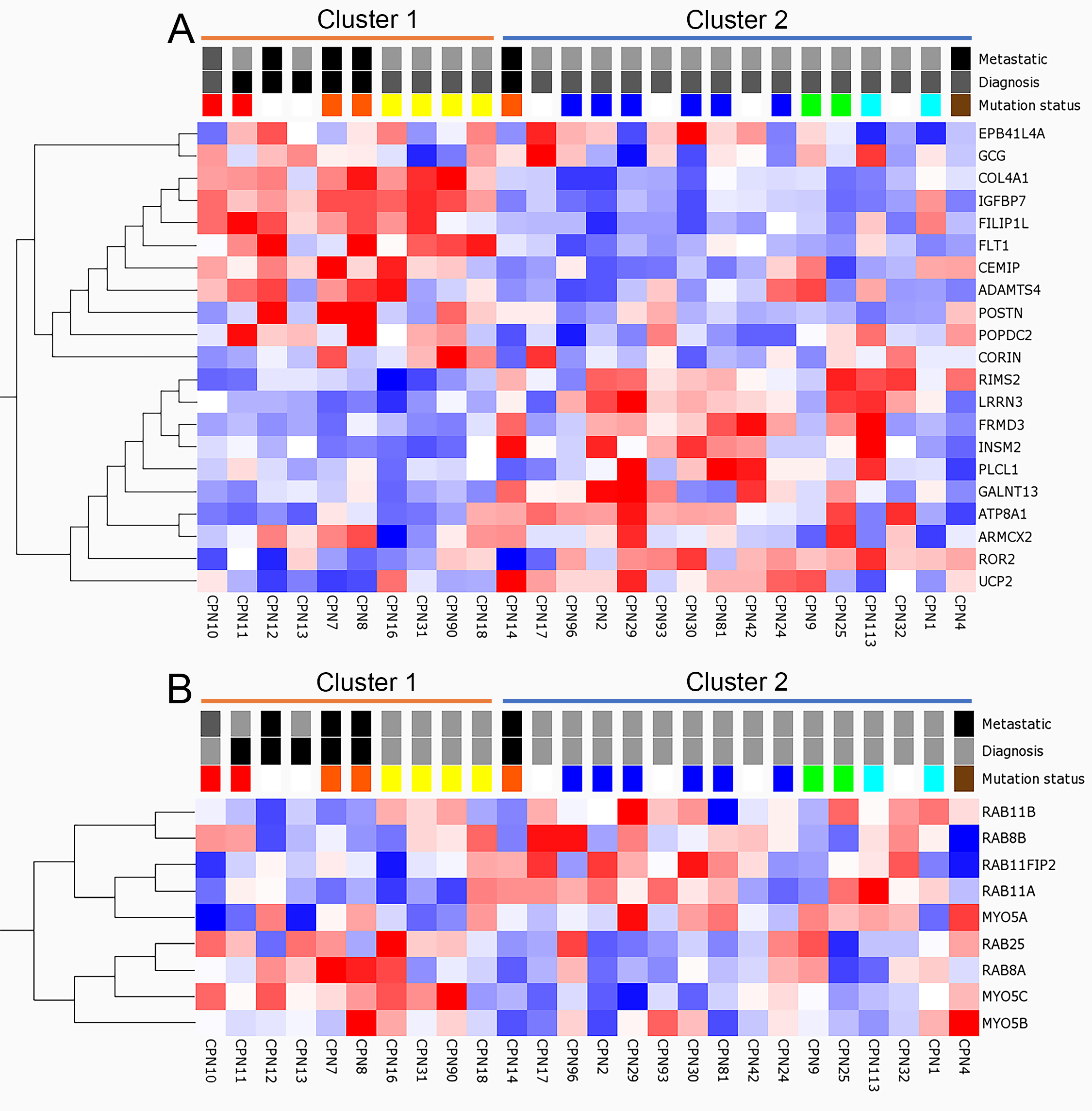

Supplement: S3 Fig — Expression of 21 out of 29 most differentially expressed genes (A) and expression of 9 MYO5B-associated RABs (B) from the functional study of three MYO5B mutants. Tumor samples are displayed according to cluster subgroups. Genes are sorted by hierarchical clustering. The heat map color scale is based on standard deviations (sd) and ranges from +2 sd (red) to -2 sd (green). The status of malignancy and diagnosis are shown by grey and black squares; black = metastatic, dark grey = multifocal, light grey = non-metastatic; black = PGL, grey = PCC. Mutation status is marked as follows: red = EPAS1, orange = SDHB, yellow = VHL, blue = RET, turquoise = NF1, green = HRAS, brown = SDHA, white = not determined (nd). (TIF) [file pgen.1008803.s003.tif]
