# Supplementary material for: MYO5B mutations in pheochromocytoma/paraganglioma promote cancer progression
Source: PLoS Genet. 2020 Jun 8;16(6):e1008803. doi: 10.1371/journal.pgen.1008803 (PMC7329139; doi:10.1371/journal.pgen.1008803)
Supplement: S1 Table — The 40 genes for calling of mutations in exomes were selected based on the following criteria; previously established or reported with germline and/or recurrent somatic mutations in PPGL (Buffet 2018 [9], Cascon 2015 [10], Dahia 2014 [7], Dwight 2017 [71], Fishbein 2017 [4], Papathomas 2014 [11], Remacha 2018 [12], Remacha 2019 [13], Yang 2015 [15], Yeh [77]), or recurrently occurring genes between lists of several PPGL studies (Castro-Vega 2015 [70], Flynn 2015 [72], Juhlin 2015 [73], Fishbein 2015 [18]), or recurrently mutated within our previous study (Wilzén 2016 [14]). Also, MYO5B-paralogs MYO5A and MYO5C were included. The 14 most commonly mutated genes in PPGL are marked with a star (*). (PDF) [file pgen.1008803.s004.pdf]

S1 Table. Gene-set for variant calling in PPGL

Gene	Inclusion criteria and reference
AHNAK2	Recurrent [14,70,72]
ARNT	Somatic [4]
ATRX*	Recurrent [14,18,70,72]
BRAF	Somatic [4]
CSDE1	Somatic [4]
DLST	Germline [13]
DNAH17	Recurrent [14,70,72]
DNMT3A	Germline [12]
EGLN1	Germline [4]
EGLN2	Germline [15]
EPAS1 *	Somatic [4]
FGFR1	Somatic [4]
FH*	Germline [7]
HRAS*	Somatic [4, 14]
IDH1	Somatic [4]
KIF1B	Germline [77]
KMT2B	Somatic [14,73]
MAML3	Somatic [4]
MAX*	Germline [4, 7]
MDH2	Germline [10]
MYCN	Recurrent [14]
MYO5A	MYO5 homolog
MYO5B	Recurrent [14]
MYO5C	MYO5 homolog
MYO9B	Recurrent [70]
NF1*	Germline & Somatic [4,7,14]
RET*	Germline & Somatic [4,7]
SDHA*	Germline [7]
SDHAF2*	Germline [7]
SDHAF3	Germline [71]
SDHB*	Germline [4, 7]
SDHC*	Germline [7]
SDHD*	Germline [4, 7]
SETD2	Somatic [4]
SLC25A11	Germline [9]
TERT	Somatic [11]
TMEM127*	Germline [4,7]
TP53	Somatic [4]
VCL	Recurrent [14]
VHL*	Germline & Somatic [4,7]

The 40 genes for calling of mutations in exomes were selected based on the following criteria; previously established or reported with germline and/or recurrent somatic mutations in PPGL (Buffet 2018 [9], Cascon 2015 [10], Dahia 2014 [7], Dwight 2017 [71], Fishbein 2017 [4], Papathomas 2014 [11], Remacha 2018 [12], Remacha 2019 [13], Yang 2015 [15], Yeh [77]), or recurrently occurring genes between lists of several PPGL studies (Castro-Vega 2015 [70], Flynn 2015 [72], Juhlin 2015 [73], Fishbein 2015 [18]), or recurrently mutated within our previous study (Wilzén 2016 [14]). Also, *MYO5B*-paralogs *MYO5A* and *MYO5C* were included. The 14 most commonly mutated genes in PPGL are marked with a star (*).
