# Supplementary material for: MYO5B mutations in pheochromocytoma/paraganglioma promote cancer progression
Source: PLoS Genet. 2020 Jun 8;16(6):e1008803. doi: 10.1371/journal.pgen.1008803 (PMC7329139; doi:10.1371/journal.pgen.1008803)
Supplement: S2 Table — Mutation analysis by Exome sequencing and MLPA (see material and methods for details). Paired tumor tissue and normal samples (T & N) or single tumor samples (T) run by different library preparation kits (SureSelect v3 or v5 or Clinical research exome (CRE v2)). Variant filtering was performed by Alissa Interpret (Agilent Technologies) and somatic filtering in paired samples (T-N) was according to Wilzen et al., 2016 [14]. Major variant: pathogenic or likely pathogenic mutation in any of the 14 PPGL susceptibility genes and their allele frequency (AF) in normal and/or tumor sample. Present in Database: Variants previously reported in ClinVar (www.ncbi.nlm.nih.gov/clinvar/) or HGMD (portal.biobase-international.com) databases. Variants were defined as germline if occurring in the normal blood/tissue sample, and as somatic if only occurring in the tumor tissue sample. Other variants: secondary variants present in the 40-gene set occurring in AF>0.2, predicted to be damaging by at least 2 out of 3 functional prediction software (Polyhen, SIFT, and MutationTaster), and present <0.1% (germline) or 0% (somatic) in normal population databases. *Variants previously reported in COSMIC were included at lower AF. nd = not determined. (PDF) [file pgen.1008803.s005.pdf]

S2 Table. Mutation analysis in 40 PPGL-associated genes

Case ID	Exome analysis	Exome pipeline	Major variant	Present in Database	AF	Germline/ Somatic	Other variants	AF	Germline/ Somatic
CPN1	Paired (T & N)	SureSelect v.3/5	NF1 NM_001042492:c.7673delA, p.E2558fs		0.2650 (T)	Somatic	MYO5A NM_000259:c.2777A>G, p.E926G	0.2933	Somatic (T)
CPN2	Paired (T & N)	SureSelect v.3/5	RET NM_020975.4: c.1900T>C, p.C634R	ClinVar	0.4875 (N)<br>0.5820 (T)	Germline			
CPN3	Paired (T & N)	SureSelect v.3/5	SDHB NM_003000.2: c.688C>T, p.R230C	ClinVar	0.5306 (N)<br>0.71111 (T)	Germline			
CPN4	Paired (T & N)	SureSelect v.3/5	SDHA NM_004168.3: c.-7A>C, p.?	HGMD	0.5 (N)<br>nd (T)	Germline			
CPN6	Paired (T & N)	SureSelect v.3/5	SDHB NM_003000.2: c.418G>T, p.V140F	ClinVar	0.4080<br>0.7523 (T)	Germline	MYCN NM_005378:c.C173C>T, p.T58M<br>VCL NM_003373:c.A692A>G, p.N231S	0.5<br>0.3259	Somatic (T)<br>Somatic (T)
CPN7	Paired (T & N)	SureSelect v.3/5	SDHB NM_003000.2: c.G600A, p.W200X	ClinVar	0.4348 (N)<br>0.7907 (T)	Germline	MYO5B NM_001080467:c.T1760C, p.L587P<br>VCL NM_003373:c.757_771del, p.253_257del*	0.2453<br>0.1889	Somatic (T)<br>Somatic (T)
CPN8	Paired (T & N)	SureSelect v.3/5	SDHB NM_003000.2: c.418G>T, p.V140F	ClinVar	0.4439 (N)<br>0.6809 (T)	Germline	SLC25A11 NM_003562.4: c.598G>T, p.A200S<br>MYCN NM_005378:c.131C>T, p.P44L*<br>MYO5B NM_001080467:c.4831G>A, p.G1611S	0.4636<br>0.0862<br>0.3103	Germline (N)<br>Somatic (T)<br>Somatic (T)
CPN9	Paired (T & N)	SureSelect v.3/5	HRAS NM_001130442:c.35G>A, p.G12D	ClinVar	0.4337 (T)	Somatic			
CPN10	Paired (T & N)	SureSelect v.3/5	EPAS1 NM_001430: c.1589C>T, p.A530V		0.4274 (T)	Somatic			
CPN11	Paired (T & N)	SureSelect v.3/5	EPAS1 NM_001430: c.1589C>T, p.A530V		0.4242 (T)	Somatic			
CPN12	Paired (T & N)	SureSelect v.3/5	SDHB deletion ex1-8 (NM_003000.2)		app. 0.25 (T)	Somatic			
CPN13	Paired (T & N)	SureSelect v.3/5	SDHB deletion ex1-8 (NM_003000.2)		app.0.45 (T)	Somatic			
CPN14	Paired (T & N)	SureSelect v.3/5	SDHB NM_003000.2: c.725G>A, p.R242H	ClinVar	0.3 (N)<br>0.7826 (T)	Germline			
CPN15	Paired (T & N)	SureSelect v.3/5	nd						
CPN123	Paired (T & N)	CREv2 clinical	SDHB NM_003000.2; c.688C>T, p.R230C	ClinVar	0.4552 (N)<br>0.8081 (T)	Germline			
CPN16	Single (T)	CREv2 clinical	VHL NM_000551.3; c.392A>G, p.N131S	HGMD	0.3436 (T)	nd			
CPN17	Single (T)	CREv2 clinical	nd						
CPN18	Single (T)	CREv2 clinical	VHL NM_000551.3: c.460C>A, p.P154T	HGMD	0.6867 (T)	nd			
CPN24	Single (T)	CREv2 clinical	RET NM_020975.4: c.2753T>C, p.M918T	ClinVar	0.3357 (T)	nd			
CPN25	Single (T)	CREv2 clinical	HRAS NM_176795.3: c.182A>G, p.Q61R	ClinVar	0.4431 (T)	nd			
CPN29	Single (T)	CREv2 clinical	RET NM_020975.4: c.1902C>G, p.C634W	ClinVar	0.3099 (T)	nd	ARNT NM_001286035.1:c.1171C>A, p.Q391K<br>MYO5A NM_000259.3:c.3581C>A, p.P1194Q	0.2272<br>0.2500	nd<br>nd
CPN30	Single (T)	CREv2 clinical	RET NM_020975.4: c.2753T>C, p.M918T	ClinVar	0.4286 (T)	nd	MYO9B NM_001130065.1 c.5176C>T, p.H1726Y	0.4589	nd
CPN31	Single (T)	CREv2 clinical	VHL NM_000551.3: c.250G>C, p.V84L	ClinVar	0.5509 (T)	nd			
CPN32	Single (T)	CREv2 clinical	SDHB deletion ex1-8 (NM_003000.2)		app.0.3 (T)	nd			
CPN42	Single (T)	CREv2 clinical	SDHB deletion ex1-8 (NM_003000.2)		app.0.5 (T)	nd			
CPN81	Single (T)	CREv2 clinical	RET NM_020975.4; c.1826G>A p.C609Y	ClinVar;<br>HGMD	0.9752 (T)	nd			
CPN90	Single (T)	CREv2 clinical	VHL deletion ex1 (NM_003000.2)		app.0.5 (T)	nd			
CPN93	Single (T)	CREv2 clinical	SDHB deletion ex1-8 (NM_003000.2)<br>NF1 deletion ex1-ex58 (NM_001042492.2)		app.0.4 (T)<br>app.0.4 (T)	nd<br>nd			
CPN96	Single (T)	CREv2 clinical	RET NM_020975.4; c.1900T>C p.C634R	ClinVar;<br>HGMD	0.5 (T)	nd			
CPN113	Single (T)	CREv2 clinical	NF1 NM_001042492.2: c.1318delC,<br>p.Arg440Efs*33	ClinVar;<br>HGMD	0.6875 (T)	nd			

Mutation analysis by Exome sequencing and MLPA (see material and methods for details). Paired tumor tissue and normal samples (T & N) or single tumors samples (T) run by different library preparation kits (SureSelect v3 or v5 or Clinical research exome (CRE v2)). Variant filtering was performed by Alissa Interpret (Agilent Technologies) and somatic filtering in paired samples (T-N) was according to Wilzen et al., 2016 [14]. Major variant: pathogenic or likely pathogenic mutation in any of the 14 PPGL susceptibility genes and their allele frequency (AF) in normal and/or tumor sample. Present in Database: Variants previously reported in ClinVar (www.ncbi.nlm.nih.gov/clinvar/) or HGMD (portal.biobase-international.com) databases. Variants were defined as germline if occurring in the normal blood/tissue sample, and as somatic if only occurring in the tumor tissue sample. Other variants: secondary variants present in the 40-gene set occurring in AF>0.2, predicted to be damaging by at least 2 out of 3 functional prediction software (Polyhen, SIFT, and MutationTaster), and present <0.1% (germline) or 0% (somatic) in normal population databases. *Variants previously reported in COSMIC were included at lower AF. nd=not determined.
