# Supplementary material for: MYO5B mutations in pheochromocytoma/paraganglioma promote cancer progression
Source: PLoS Genet. 2020 Jun 8;16(6):e1008803. doi: 10.1371/journal.pgen.1008803 (PMC7329139; doi:10.1371/journal.pgen.1008803)
Supplement: S3 Table — The 153 genes with highest variance in 26 tumors samples, discriminating two expression clusters of PPGL tumors. (PDF) [file pgen.1008803.s006.pdf]

S3 Table. Gene-set for expression clustering of PPGL tumors.

GeneSymbol	GeneName
A2BP1	ataxin 2-binding protein 1
ABCC8	ATP-binding cassette, sub-family C (CFTR/MRP), member 8
ACTG2	actin, gamma 2, smooth muscle, enteric
ACVR1C	activin A receptor, type IC
ADCYAP1	adenylate cyclase activating polypeptide 1 (pituitary)
AGRP	agouti related protein homolog (mouse)
AK3L1	adenylate kinase 3-like 1
ANGPTL4	angiopoietin-like 4
ANK3	ankyrin 3, node of Ranvier (ankyrin G)
APOLD1	apolipoprotein L domain containing 1
AREG	amphiregulin (schwannoma-derived growth factor)
BCAT1	branched chain aminotransferase 1, cytosolic
BCHE	butyrylcholinesterase
C1R	complement component 1, r subcomponent
C1S	complement component 1, s subcomponent
C20orf103	chromosome 20 open reading frame 103
C6orf176	chromosome 6 open reading frame 176
C7orf16	chromosome 7 open reading frame 16
CACNA1B	calcium channel, voltage-dependent, N type, alpha 1B subunit
CCL2	chemokine (C-C motif) ligand 2
CD24	CD24 molecule
CGNL1	cingulin-like 1
CNN1	calponin 1, basic, smooth muscle
COL27A1	collagen, type XXVII, alpha 1
CRH	corticotropin releasing hormone
CRTAC1	cartilage acidic protein 1
CTGF	connective tissue growth factor
CXCR7	chemokine (C-X-C motif) receptor 7
CYP26A1	cytochrome P450, family 26, subfamily A, polypeptide 1
CYR61	cysteine-rich, angiogenic inducer, 61
DCN	decorin
DDX3Y	DEAD (Asp-Glu-Ala-Asp) box polypeptide 3, Y-linked
DES	desmin
DIRAS3	DIRAS family, GTP-binding RAS-like 3
DLK1	delta-like 1 homolog (Drosophila)
DRD1IP	dopamine receptor D1 interacting protein
ECEL1	endothelin converting enzyme-like 1
EFEMP1	EGF-containing fibulin-like extracellular matrix protein 1
EGLN3	egl nine homolog 3 (C. elegans)
EGR1	early growth response 1
EGR2	early growth response 2 (Krox-20 homolog, Drosophila)
EGR4	early growth response 4
EMP1	epithelial membrane protein 1
FABP6	fatty acid binding protein 6, ileal (gastrotropin)
FAM19A3	family with sequence similarity 19 (chemokine (C-C motif)-like), member A3
FAM19A4	family with sequence similarity 19 (chemokine (C-C motif)-like), member A4
FBLN1	fibulin 1
FHL2	four and a half LIM domains 2
FHOD3	formin homology 2 domain containing 3
FMOD	fibromodulin
FOS	v-fos FBJ murine osteosarcoma viral oncogene homolog
FOSB	FBJ murine osteosarcoma viral oncogene homolog B
FSTL5	follistatin-like 5
GABRG2	gamma-aminobutyric acid (GABA) A receptor, gamma 2
GAL	galanin
GALNT14	UDP-N-acetyl-alpha-D-galactosamine:polypeptide N-acetylgalactosaminyltransferase 14 (GalNAc-T14)
GCGR	glucagon receptor
GDF10	growth differentiation factor 10
GLDN	gliomedin
GNG11	guanine nucleotide binding protein (G protein), gamma 11
GNG8	guanine nucleotide binding protein (G protein), gamma 8
GPR30	G protein-coupled receptor 30
GRID2	glutamate receptor, ionotropic, delta 2
GRIK1	glutamate receptor, ionotropic, kainate 1
H19	H19, imprinted maternally expressed untranslated mRNA
HK2	hexokinase 2
HOXA9	homeobox A9
HOXB5	homeobox B5
HOXB6	homeobox B6
HSD3B2	hydroxy-delta-5-steroid dehydrogenase, 3 beta- and steroid delta-isomerase 2
HSPB7	heat shock 27kDa protein family, member 7 (cardiovascular)
IGF2	insulin-like growth factor 2 (somatomedin A)
IGFBP2	insulin-like growth factor binding protein 2, 36kDa
IGH@	immunoglobulin heavy locus
IL13RA2	interleukin 13 receptor, alpha 2
INHBB	inhibin, beta B (activin AB beta polypeptide)
ITGA7	integrin, alpha 7

JAKMIP1	janus kinase and microtubule interacting protein 1
KBTBD11	kelch repeat and BTB (POZ) domain containing 11
KCNE3	potassium voltage-gated channel, Isk-related family, member 3
KIAA0125	KIAA0125
KIRREL3	kin of IRRE like 3 (Drosophila)
LAYN	layilin
LOC202134	hypothetical protein LOC202134
LOC283953	hypothetical LOC283953
LOC285878	hypothetical protein LOC285878
LOC642652	hypothetical LOC642652
LOC730130	hypothetical protein LOC730130
MEG3	maternally expressed 3
MOXD1	monooxygenase, DBH-like 1
MST150	MSTP150
NEFH	neurofilament, heavy polypeptide 200kDa
NGB	neuroglobin
NLF2	nuclear localized factor 2
NNMT	nicotinamide N-methyltransferase
NOS2A	nitric oxide synthase 2A (inducible, hepatocytes)
NOV	nephroblastoma overexpressed gene
NPTX2	neuronal pentraxin II
NPY	neuropeptide Y
NR4A3	nuclear receptor subfamily 4, group A, member 3
NTNG2	netrin G2
NXPH1	neurexophilin 1
NY-REN-7	NY-REN-7 antigen
OSAP	ovary-specific acidic protein
PCDH11Y	protocadherin 11 Y-linked
PCP4	Purkinje cell protein 4
PCSK1	proprotein convertase subtilisin/kexin type 1
PCSK2	proprotein convertase subtilisin/kexin type 2
PDZK1	PDZ domain containing 1
PENK	proenkephalin
PNMT	phenylethanolamine N-methyltransferase
PON3	paraoxonase 3
PROK2	prokineticin 2
PTN	pleiotrophin (heparin binding growth factor 8, neurite growth-promoting factor 1)
RAB34	RAB34, member RAS oncogene family
RARRES2	retinoic acid receptor responder (tazarotene induced) 2
RASGEF1A	RasGEF domain family, member 1A
RET	ret proto-oncogene
RFXDC1	regulatory factor X domain containing 1
RGN	regucalcin (senescence marker protein-30)
RP13-102H20.1	hypothetical protein FLJ30058
RPS4Y1	ribosomal protein S4, Y-linked 1
RPS4Y2	ribosomal protein S4, Y-linked 2
RSPO1	R-spondin homolog (Xenopus laevis)
SALL4	sal-like 4 (Drosophila)
SAMD5	sterile alpha motif domain containing 5
SCGN	secretagogin, EF-hand calcium binding protein
SFRP1	secreted frizzled-related protein 1
SHC3	SHC (Src homology 2 domain containing) transforming protein 3
SHD	Src homology 2 domain containing transforming protein D
SLC16A12	solute carrier family 16, member 12 (monocarboxylic acid transporter 12)
SLC32A1	solute carrier family 32 (GABA vesicular transporter), member 1
SLC35F3	solute carrier family 35, member F3
SLC6A2	solute carrier family 6 (neurotransmitter transporter, noradrenalin), member 2
SLITRK6	SLIT and NTRK-like family, member 6
SMOC1	SPARC related modular calcium binding 1
SORCS3	sortilin-related VPS10 domain containing receptor 3
SORL1	sortilin-related receptor, L(DLR class) A repeats-containing
SPON1	spondin 1, extracellular matrix protein
SPP1	secreted phosphoprotein 1 (osteopontin, bone sialoprotein I, early T-lymphocyte activation 1)
SST	somatostatin
STAR	steroidogenic acute regulatory protein
STRA6	stimulated by retinoic acid gene 6 homolog (mouse)
SV2B	synaptic vesicle glycoprotein 2B
SV2C	synaptic vesicle glycoprotein 2C
SYNPR	synaptoporin
SYT13	synaptotagmin XIII
TAC3	tachykinin 3 (neuromedin K, neurokinin beta)
TFPI2	tissue factor pathway inhibitor 2
THBS2	thrombospondin 2
TM4SF4	transmembrane 4 L six family member 4
XIST	X (inactive)-specific transcript
ZFP36	zinc finger protein 36, C3H type, homolog (mouse)

The 153 genes with highest variance in 26 tumors samples, discriminating two expression clusters of PPGL tumors.
