# Supplementary material for: MYO5B mutations in pheochromocytoma/paraganglioma promote cancer progression
Source: PLoS Genet. 2020 Jun 8;16(6):e1008803. doi: 10.1371/journal.pgen.1008803 (PMC7329139; doi:10.1371/journal.pgen.1008803)
Supplement: S4 Table — Microarray expression analysis of SK-N-AS constructs; MUT 1(p.L587P), MUT 2 (p.G1611S), and MUT 3 (p.R1641C) and wild type (WT) MYO5B compared to empty vector (EV). Calculations are based on measures from two SK-N-AS passages (p23 and p30) for each mutation and time point of proliferation (24h, 48h and 72h). t-statistic (t), significance (P.Value, and adj.p) and fold change (FC) from MUTvsEV group comparison. (PDF) [file pgen.1008803.s007.pdf]

S4 Table. MYO5B microarray mRNA expression of three MYO5B mutants versus empty vector

Comparison	File name	logFC	FC	AveExpr	t	P.Value	adj.p	Gene symbo	Gene name
MUT1vsEV_24_group	TC1800009290.hg.1	1,636725607	3,109592659	7,0390488	14,5019261	1,80E-05	0,01119896	MYO5B	myosin VB
MUT1vsEV_48_group	TC1800009290.hg.1	1,053797988	2,075987833	6,36332475	1,86322822	0,1116624	0,72152826	MYO5B	myosin VB
MUT1vsEV_72_group	TC1800009290.hg.1	1,451081137	2,734128666	6,58910504	4,07387388	0,00832155	0,2725483	MYO5B	myosin VB
MUT2vsEV_24_group	TC1800009290.hg.1	1,38612645	2,613759589	6,91374922	14,0882487	1,52E-05	0,00785222	MYO5B	myosin VB
MUT2vsEV_48_group	TC1800009290.hg.1	2,138077606	4,401751212	6,90546456	8,19714745	0,00031448	0,32386982	MYO5B	myosin VB
MUT2vsEV_72_group	TC1800009290.hg.1	2,220118587	4,659317317	6,97362377	4,60738119	0,00527745	0,36221581	MYO5B	myosin VB
MUT3vsEV_24_group	TC1800009290.hg.1	0,635143379	1,553092093	6,53825769	6,45286685	0,00103022	0,0729943	MYO5B	myosin VB
MUT3vsEV_48_group	TC1800009290.hg.1	0,561094104	1,47538769	6,11697281	2,05107356	0,08514124	0,87948601	MYO5B	myosin VB
MUT3vsEV_72_group	TC1800009290.hg.1	0,890159551	1,853381082	6,30864425	2,88890243	0,03300276	0,39488784	MYO5B	myosin VB
WTvsEV_24_group	TC1800009290.hg.1	0,771781039	1,707376277	6,60657652	4,73264477	0,00157534	0,12696861	MYO5B	myosin VB
WTvsEV_48_group	TC1800009290.hg.1	0,70779808	1,633309368	6,1903248	5,55612038	0,00173524	0,24266756	MYO5B	myosin VB
WTvsEV_72_group	TC1800009290.hg.1	0,878400227	1,83833568	6,30276459	3,79944637	0,00927524	0,27147469	MYO5B	myosin VB

Microarray expression analysis of SK-N-AS constructs; MUT 1(p.L587P), MUT 2 (p.G1611S), and MUT 3 (p.R1641C) and wild type (WT) MYO5B compared to empty vector (EV). Calculations are based on measures from two SK-N-AS passages (p23 and p30) for each mutation and time point of proliferation (24h, 48h and 72h). t-statistic (t), significance (P.Value, and adj.p) and fold change (FC) from MUTvsEV group comparison.
