# Supplementary material for: MYO5B mutations in pheochromocytoma/paraganglioma promote cancer progression
Source: PLoS Genet. 2020 Jun 8;16(6):e1008803. doi: 10.1371/journal.pgen.1008803 (PMC7329139; doi:10.1371/journal.pgen.1008803)
Supplement: S6 Table — The top-ranked differentially expressed genes (see S5 Table) from all three MYO5B mutants were analyzed using GOrilla (Gene Ontology enRIchment anaLysis and visuaLizAtion tool, http://cbl-gorilla.cs.technion.ac.il) with all genes present on the microarray used as background. N = total no. of genes recognized by Gorilla and associated with GO terms, B = no. of genes associated with the specified GO term, n = no. of genes recognized by GOrilla in the target set (one gene annotation including several genes and one gene not associated with any GO terms were excluded), b = no. of genes in the target set associated with the specified GO term. P-value threshold < 10^-3. (PDF) [file pgen.1008803.s009.pdf]

S6 Table. Gene ontology enrichment analysis on top differentially expressed genes

GO Term	Description	P-value	Enrichment	N	B	n	b	Genes
GO:0030335	positive regulation of cell migration	7.62E-6	7.61	17280	478	38	8	ATP8A1, LPAR1, FN1, SEMA3A, FLT1, CEMIP, ROR2, POSTN
GO:2000147	positive regulation of cell motility	1.09E-5	7.25	17280	502	38	8	ATP8A1, LPAR1, FN1, SEMA3A, FLT1, CEMIP, ROR2, POSTN
GO:0051272	positive regulation of cellular component movement	1.33E-5	7.05	17280	516	38	8	ATP8A1, LPAR1, FN1, SEMA3A, FLT1, CEMIP, ROR2, POSTN
GO:0040017	positive regulation of locomotion	1.59E-5	6.88	17280	529	38	8	ATP8A1, LPAR1, FN1, SEMA3A, FLT1, CEMIP, ROR2, POSTN
GO:0030334	regulation of cell migration	4.49E-5	5.12	17280	799	38	9	LPAR1, ATP8A1, FN1, IL24, SEMA3A, FLT1, CEMIP, ROR2, POSTN
GO:1900020	positive regulation of protein kinase C activity	4.69E-5	181.89	17280	5	38	2	CEMIP, ROR2
GO:1900019	regulation of protein kinase C activity	4.69E-5	181.89	17280	5	38	2	CEMIP, ROR2
GO:0021889	olfactory bulb interneuron differentiation	4.69E-5	181.89	17280	5	38	2	SALL1, DLX5
GO:1901166	neural crest cell migration involved in autonomic nervous system development	7.02E-5	151.58	17280	6	38	2	FN1, SEMA3A
GO:0021675	nerve development	7.85E-5	35.90	17280	38	38	3	SALL1, LPAR1, SEMA3A
GO:2000145	regulation of cell motility	7.87E-5	4.76	17280	859	38	9	LPAR1, ATP8A1, FN1, IL24, SEMA3A, FLT1, CEMIP, ROR2, POSTN
GO:0040012	regulation of locomotion	1.38E-4	4.43	17280	924	38	9	LPAR1, ATP8A1, FN1, IL24, SEMA3A, FLT1, CEMIP, ROR2, POSTN
GO:0051270	regulation of cellular component movement	1.46E-4	4.40	17280	931	38	9	LPAR1, ATP8A1, FN1, IL24, SEMA3A, FLT1, CEMIP, ROR2, POSTN
GO:0021782	glial cell development	1.58E-4	28.42	17280	48	38	3	PHGDH, LPAR1, ROR2
GO:0032879	regulation of localization	2.21E-4	2.62	17280	2604	38	15	FN1, SEMA3A, CEMIP, FLT1, ROR2, LYPD1, ATP8A1, LPAR1, IL24, PLCL1, RIMS2, GCG, CORIN, POSTN, SCN8A
GO:0021879	forebrain neuron differentiation	4.21E-4	64.96	17280	14	38	2	SALL1, DLX5
GO:0030198	extracellular matrix organization	5.89E-4	7.24	17280	314	38	5	ADAMTS4, ITGA10, FN1, COL4A1, POSTN
GO:0048588	developmental cell growth	7.17E-4	17.05	17280	80	38	3	SORBS2, SEMA3A, POSTN
GO:0016049	cell growth	7.71E-4	16.64	17280	82	38	3	SORBS2, SEMA3A, POSTN
GO:0051962	positive regulation of nervous system development	8.02E-4	5.34	17280	511	38	6	LPAR1, FN1, SEMA3A, RIMS2, LRRN3, ROR2

The top-ranked differentially expressed genes (see S5 Table) from all three MYO5B mutants were analyzed using GOrilla (Gene Ontology enRIchment anaLysis and visuaLizAtion tool, http://cbl-gorilla.cs.technion.ac.il) with all genes present on the microarray used as background. N=total no. of genes recognized by Gorilla and associated with GO terms, B=no. of genes associated with the specified GO term, n=no. of genes recognized by GOrilla in the target set (one gene annotation including several genes and one gene not associated with any GO terms were excluded), b=no. of genes in the target set associated with the specified GO term. P-value threshold < 10^-3.
