# Supplementary material for: MYO5B mutations in pheochromocytoma/paraganglioma promote cancer progression
Source: PLoS Genet. 2020 Jun 8;16(6):e1008803. doi: 10.1371/journal.pgen.1008803 (PMC7329139; doi:10.1371/journal.pgen.1008803)
Supplement: S7 Table — Gene Set Enrichment Analysis (GSEA) performed on expression gene lists (ranked after mean fold change) for each mutant; MUT1 (p.L587P), MUT2 (p.G1611S), and MUT3 (p.R1641C), using 236 gene sets (50 H Hallmark, and 186 C2: KEGG curated gene sets) from the Molecular Signature database (http://software.broadinstitute.org/gsea/msigdb/index.jsp). The overlapping gene sets among the 20 top-ranked with up-regulated genes (UP) and 20 top-ranked with down-regulated genes (DOWN) for each mutations are listed. (PDF) [file pgen.1008803.s010.pdf]

S7 Table. Gene set enrichment analysis (GSEA) of ranked gene lists

UP

**Name**	**Genes (tot)**	**Mutation**	**Stat (dist.dir)**	**p (dist.dir.up)**	**p adj (dist.dir.up)**	**p (dist.dir.dn)**	**p adj (dist.dir.dn)**	**Genes (up)**	**Genes (down)**	**Rank**
HALLMARK_MYC_TARGETS_V1	200	MUT2	0,38295	0,0009058	0,16153	NA	NA	138	62	4
		MUT1	0,43856	0,00021436	0,056484	NA	NA	147	53	10
KEGG_BASE_EXCISION_REPAIR	33	MUT1	0,65146	0,0014236	0,025126	NA	NA	28	5	6
		MUT2	0,50365	0,039056	0,18487	NA	NA	23	10	9
KEGG_BASAL_TRANSCRIPTION_FACTORS	35	MUT2	0,58108	0,0052214	0,14737	NA	NA	28	7	1
		MUT3	0,32971	0,35469	0,95288	NA	NA	26	9	15
HALLMARK_E2F_TARGETS	200	MUT1	0,57294	0,00021436	0	NA	NA	170	30	1
		MUT3	0,22234	0,5425	0,96653	NA	NA	115	85	16
KEGG_RENIN_ANGIOTENSIN_SYSTEM	17	MUT1	0,73524	0,0022481	0,018168	NA	NA	11	6	2
		MUT3	0,28159	0,77244	0,98051	NA	NA	7	10	19
KEGG_TERPENOID_BACKBONE_BIOSYNTHESIS	15	MUT1	0,70412	0,013427	0,052002	NA	NA	12	3	8
		MUT2	0,64099	0,033571	0,19602	NA	NA	13	2	13
KEGG_GLYCOSAMINOGLYCAN_DEGRADATION	21	MUT3	0,37234	0,37081	0,90174	NA	NA	9	12	6
		MUT1	0,55521	0,064542	0,18881	NA	NA	10	11	18
KEGG_DRUG_METABOLISM_OTHER_ENZYMES	51	MUT3	0,27357	0,53683	0,97784	NA	NA	27	24	18
		MUT1	0,46417	0,03798	0,19044	NA	NA	28	23	19

DOWN

**Name**	**Genes (tot)**	**Mutation**	**Stat (dist.dir)**	**p (dist.dir.up)**	**p adj (dist.dir.up)**	**p (dist.dir.dn)**	**p adj (dist.dir.dn)**	**Genes (up)**	**Genes (down)**	**Rank**
HALLMARK_TNFA_SIGNALING_VIA_NFKB	200	MUT1	-0,57065	NA	NA	0,00018737	0	69	131	1
		MUT3	-0,58875	NA	NA	0,00014908	0	59	141	1
HALLMARK_INFLAMMATORY_RESPONSE	199	MUT1	-0,47456	NA	NA	0,0001912	0,02152	82	117	3
		MUT3	-0,46823	NA	NA	0,00015113	0,029599	74	125	6
KEGG_ECM_RECEPTOR_INTERACTION	84	MUT3	-0,54652	NA	NA	0,00049172	0,015786	30	54	3
		MUT1	-0,54317	NA	NA	0,00019478	0,034618	30	54	7
KEGG_ARRHYTHMOGENIC_RIGHT_VENTRICULAR_CARDIOMYOPATHY_ARVC	74	MUT1	-0,55136	NA	NA	0,00038647	0,024958	29	45	5
	74	MUT3	-0,5188	NA	NA	0,00099536	0,040185	29	45	8
HALLMARK_COAGULATION	138	MUT3	-0,48868	NA	NA	0,00015903	0,024082	66	72	4
		MUT1	-0,44516	NA	NA	0,00096376	0,094573	53	85	11
HALLMARK_EPITHELIAL_MESENCHYMAL_TRANSITION	200	MUT3	-0,49226	NA	NA	0,00014908	0,013229	93	107	2
		MUT1	-0,41111	NA	NA	0,0013116	0,11322	91	109	14
HALLMARK_KRAS_SIGNALING_UP	199	MUT1	-0,47205	NA	NA	0,0001912	0,021304	80	119	2
		MUT3	-0,42483	NA	NA	0,00015113	0,061088	89	110	14
KEGG_HYPERTROPHIC_CARDIOMYOPATHY_HCM	83	MUT1	-0,54171	NA	NA	0,00019268	0,029393	39	44	6
KEGG_HYPERTROPHIC_CARDIOMYOPATHY_HCM	83	MUT3	-0,49004	NA	NA	0,0018531	0,04927	34	49	11
KEGG_SMALL_CELL_LUNG_CANCER	84	MUT3	-0,52473	NA	NA	0,00065563	0,025757	34	50	5
		MUT1	-0,47299	NA	NA	0,0054538	0,11151	42	42	13
KEGG_DILATED_CARDIOMYOPATHY	90	MUT1	-0,54353	NA	NA	0,00019264	0,038623	41	49	8
		MUT3	-0,4458	NA	NA	0,0062418	0,081967	43	47	18
KEGG_COMPLEMENT_AND_COAGULATION_CASCADES	69	MUT3	-0,49684	NA	NA	0,0041722	0,058933	28	41	13
		MUT1	-0,4852	NA	NA	0,0080568	0,11629	23	46	15
HALLMARK_COMPLEMENT	200	MUT3	-0,43853	NA	NA	0,00014908	0,045257	75	125	10
		MUT1	-0,3914	NA	NA	0,0026232	0,18165	89	111	19
KEGG_PATHWAYS_IN_CANCER	325	MUT1	-0,39152	NA	NA	0,00037147	0,10864	154	171	12
		MUT3	-0,39123	NA	NA	0,00014337	0,080099	144	181	17

Gene Set Enrichment Analysis (GSEA) performed on expression gene lists (ranked after mean fold change) for each mutant; MUT1 (p.L587P), MUT2 (p.G1611S), and MUT3 (p.R1641C), using 236 gene sets (50 H Hallmark, and 186 C2: KEGG curated gene sets) from the Molecular Signature database (http://software.broadinstitute.org/gsea/msigdb/index.jsp). The overlapping gene sets among the 20 top-ranked with up-regulated genes (UP) and 20 top-ranked with down-regulated genes (DOWN) for each mutations are listed.
