# Supplementary material for: MYO5B mutations in pheochromocytoma/paraganglioma promote cancer progression
Source: PLoS Genet. 2020 Jun 8;16(6):e1008803. doi: 10.1371/journal.pgen.1008803 (PMC7329139; doi:10.1371/journal.pgen.1008803)
Supplement: S8 Table — Assay-ID and target information for TaqMan primers and probes used for qPCR verification of the 29 most differentially expressed genes in MYO5B mutants. INV = Inventoried assay, MTO = Made-to-order assay. (PDF) [file pgen.1008803.s011.pdf]

S8 Table. TaqMan Array Micro Fluidic Cards design

Assay ID	Availability	Gene Symbol(s)	Gene Name(s)	Gene Alias(es)	RefSeq(s)	GenBank mRNA(s)	Amplicon Length (bp)
Hs00261625_s1	INV	INSM2	INSM transcriptional repressor 2	IA-6;IA6;mlt1	NM_032594.3	AB037912.1;AK096639.1;BC130377.1;JX403968.1;AF260323.1;BC130379.1	98
Hs00287613_m1	INV	GALNT13	polypeptide N-acetylgalactosaminyltransferase 13	GalNAc-T13	XM_017003261.1;XM_017003260.1;XM_011510538.2;XM_017003263.1;XM_017003262.1;XM_017003259.1;XM_017003258.1;NM_001301627.1;NM_052917.3;XM_011510537.2	AJ505991.2;BC101034.2;BC101031.1;AB067505.1;AB078142.1;KJ535058.1;AK131195.1;BC101033.1;BC101032.2;AK308675.1;KJ534843.1	62
Hs01075227_m1	INV	UCP2	uncoupling protein 2	BMIQ4;SLC25A8;UCPH	NM_003355.2	AK025742.1;AK222540.1;AK222557.1;U94592.1;U82819.1;BC011737.2	62
Hs99999905_m1	INV	GAPDH	glyceraldehyde-3-phosphate dehydrogenase	G3PD, GAPD, HEL-S-162eP	NM_001289746.1;NM_002046.5	AB062273.1;AF261085.1;AK026525.1;AK299448.1;AK308198.1;AY007133.1;BC001601.1;BC004109.2;BC009081.1;BC013310.2;BC020308.1;BC025925.1;BC026907.1;BC029340.1;BC029618.1;BC083511.1;BU155402.1;EU668321.1;HY022295.1;M17851.1;M33197.1;X53778.1	122
Hs00198141_m1	INV	CORIN	corin, serine peptidase	ATC2;CRN;Lrp4;PEE5;TMPRSS10	NM_001278586.1;NM_001278585.1;NM_006587.3	BC110451.2;AK302816.1;AK304038.1;AF133845.1;AK304466.1;AK297675.1	102
Hs00604157_m1	INV	FRMD3	FERM domain containing 3	4.1O;EPB41L4O;EPB41LO;P410	XM_017014588.1;NM_174938.5;XM_017014589.1;XM_017014590.1;NM_001244960.1;NM_001244959.1;NM_001244961.1	BC037253.1;AK223597.1;EF560742.1;AK297722.1;AK094281.1;AY137774.1	80
Hs01932946_s1	INV	ARMCX2	armadillo repeat containing, X-linked 2	ALEX2;GASP9	XM_017029988.1;XM_017029989.1;XM_017029987.1;XM_005278117.1;XM_017029992.1;XM_005278116.1;XM_017029990.1;XM_017029991.1;NM_001282231.1;XM_017029994.1;NM_014782.6;XM_011531071.1;NM_177949.3;XM_011531072.1;XM_017029993.1;XM_005278113.1;XM_017029997.1;XM_005278115.1;XM_017029995.1;XM_005278114.1;XM_017029996.1;XM_005278109.1;XM_005278111.1;XM_005278110.1	AB011084.1;AK291342.1;BX648494.1;BC012541.1;BC015926.1	148
Hs00196518_m1	INV	PLCL1	phospholipase C like 1	PLCE;PLCL;PLDL1;PPP1R127;PRIP	XM_005246643.3;XM_017004339.1;NM_006226.3;XM_017004340.1;XM_011511351.2;XM_005246644.3	BX537442.1;AK127514.1;D42108.1;BC101531.1;BC111985.1;AK302673.1	65
Hs01031536_m1	INV	GCG	glucagon	GLP1;GLP2;GRPP	NM_002054.4	BC005278.1;BM272442.1;J04040.1;BT006813.1	86
Hs04384957_m1	INV	FILIP1L	filamin A interacting protein 1 like	DOC-1;DOC1;GIP130;GIP90	NM_001042459.2;NM_182909.3	AX776298.1;AF514867.1;AF514868.1;AF514869.1;BC020941.1;BC017987.1;AY642382.1;AF329092.1;AX776292.1;AX776296.1;AX776294.1	84
Hs00192708_m1	INV	ADAMTS4	ADAM metallopeptidase with thrombospondin type 1 motif 4	ADAMTS-2;ADAMTS-4;ADMP-1	NM_005099.5;NM_001320336.1	AB014588.1;AY358886.1;AF148213.1;BC063293.1;AK291573.1	63
Hs00173810_m1	INV	SEMA3A	semaphorin 3A	COLL1;HH16;Hsema-I;Hsema-III;SEMA1;SEMAD;SEMAIII;SEMAL;SemD;coll-1	XM_006715839.3;NM_006080.2;XM_005250110.3;XM_011515734.2;XM_017011673.1;XM_005250111.4	BC111416.1;L26081.1;AK289954.1	80
Hs01052961_m1	INV	FLT1	fms related tyrosine kinase 1	FLT;FLT-1;VEGFR-1;VEGFR1	XM_011535014.1;NM_001160031.1;NM_001160030.1;NM_001159920.1;XM_017020485.1;NM_002019.4	AK292936.1;AF063657.2;EU368830.1;AK300392.1;EU360600.1;U01134.1;AK309901.1;EU826561.1;BC039007.1	72
Hs01552114_m1	INV	CEMIP	cell migration inducing hyaluronan binding protein	CCSP1;HYBID;KIAA1199;TMEM2L	NM_001293298.1;NM_001293304.1;NM_018689.2	AB103330.1;AY581148.1;AY585237.1;AY581149.1;AY007811.1;BC020256.1;AL359061.1;AB033025.1;AK026540.1	61
Hs00266237_m1	INV	COL4A1	collagen type IV alpha 1 chain	BSVD;RATOR	NM_001845.5;XM_011521048.2;NM_001303110.1	X05561.1;BC151220.1;AB209646.1;Y00706.1;BC142626.1	75
Hs00266026_m1	INV	IGFBP7	insulin like growth factor binding protein 7	AGM;FSTL2;IBP-7;IGFBP-7;IGFBP-7v;IGFBPRP1;MAC25;PSF;RAMSVPS;TAF	NM_001553.2;NM_001253835.1	AF540057.1;L19182.1;S75725.1;BC066339.1;BT006654.1;AK316082.1;AK303915.1;BC017201.2	86

Hs01573641_mH	MTO	DLX5	distal-less homeobox 5	SHFM1D	XM_017011803.1;NM_005221.5;XM_005250185.3	AK023493.1;BC006226.2;BT006903.1	116
Hs00904660_m1	INV	EMB	embigin	GP70	XM_011543146.2;NM_198449.2	DC309453.1;BC059398.1;AK300860.1;AK304226.1	150
Hs01566750_m1	INV	POSTN	periostin	OSF-2;OSF2;PDLPOSTN;PN	XM_017020356.1;NM_001135936.1;NM_001286667.1;NM_001135935.1;XM_017020355.1;NM_006475.2;NM_001135934.1;XM_005266231.2;NM_001286666.1;NM_001286665.1;XM_005266232.2	AY140646.1;BC106709.1;EU262886.1;AK300261.1;EU262884.1;EU262885.1;BC106710.1;BY795054.2;AY918092.1;GU354210.1;D13665.1;EU262883.1;D13666.1	67
Hs01106329_m1	INV	PHGDH	phosphoglycerate dehydrogenase	3-PGDH;3PGDH;HEL-S-113;NLS;NLS1;PDG;PGAD;PGD;PGDH;PHGDHD;SERA	XM_011541228.1;XM_011541227.2;XM_011541226.1;XM_011541231.2;NM_006623.3	FJ224347.1;AK309662.1;AK315360.1;BC023235.1;CR456795.1;AK129762.1;AK093306.1;BC032110.2;AF171237.1;AF171236.1;BC011262.1;BC001349.2;AF171235.1;BC000303.2	58
Hs01102345_m1	INV	RPL37A	ribosomal protein L37a	L37A	NM_000998.4	L22154.1;L06499.1;CD249666.1;X66699.1;BC067789.1;BC082239.1;AK289472.1;BC000555.2;BC014262.2;BC016748.2	125
Hs00427620_m1	INV	TBP	TATA-box binding protein	GTF2D;GTF2D1;HDL4;SCA17;TFIID	NM_001172085.1;NM_003194.4	BC109054.1;CR456776.1;Z22828.1;X54993.1;AK315222.1;BT019657.1;BC109053.1;BC110341.1;M34960.1;AK304648.1;M55654.1	91
Hs01597483_m1	INV	ATP8A1	ATPase phospholipid transporting 8A1	ATPASEII;ATPIA;ATPP2	XM_011513618.1;XM_011513615.1;XM_017007646.1;NM_006095.2;XM_005248043.2;XM_017007645.1;XM_011513616.2;XM_017007647.1;NM_001105529.1	AB209687.1;AF067820.1;AB013452.1;BC020943.1;BP232466.1;BC109317.1;BC109318.1	77
Hs00896176_m1	INV	ROR2	receptor tyrosine kinase like orphan receptor 2	BDB;BDB1;NTRKR2	XM_005252008.4;XM_017014762.1;XM_006717121.3;XM_017014763.1;NM_004560.3;XM_005252009.3	BC130522.1;BC033697.1;M97639.1;AB209154.1	68
Hs00230170_m1	INV	AKR1E2	aldo-keto reductase family 1 member E2	AKR1CL2;AKRDC1;LoopADR;hTSP;htAKR	XM_011519718.2;XM_011519720.2;XM_011519724.2;XM_017016743.1;NR_073125.1;XM_017016744.1;XM_011519722.2;NR_073126.1;XM_017016745.1;NR_073127.1;XM_011519729.2;NM_001040177.2;XM_011519719.2;XM_011519725.2;NM_001271021.1;NM_001271025.1;XM_011519715.2	DA006578.1;BC002862.2;AF263242.1;AB055603.1;AB040820.1;AB040821.1;AB040822.1;AK074211.1	65
Hs00998517_m1	INV	ACSS3	acyl-CoA synthetase short-chain family member 3	-	XM_011538740.2;XM_005269150.2;XM_005269151.4;NM_024560.2	BC015769.1;AK025616.1;AK074938.1;BC009317.2;AX764480.1;AX764482.1	74
Hs01548765_m1	INV	SALL1	spalt like transcription factor 1	HEL-S-89;HSAL1;Sal-1;TBS;ZNF794	XM_006721241.3;XM_011523254.2;NM_001127892.1;NM_002968.2	EU668343.1;Y18265.1;AK295103.1	78
Hs00223297_m1	INV	EPB41L4A	erythrocyte membrane protein band 4.1 like 4A	EPB41L4;NBL4	XM_011543531.2;NM_022140.3;XM_011543530.1;XM_017009689.1;XM_011543533.1;XM_011543532.1	BC114632.1;AB030240.1;BC114942.1	68
Hs00988745_m1	MTO	RIMS2	regulating synaptic membrane exocytosis 2	OBOE;RAB3IP3;RIM2	XM_017014026.1;XM_006716698.3;XM_017014024.1;XM_017014030.1;XM_017014028.1;XM_017014018.1;XM_017014016.1;XM_017014022.1;XM_017014020.1;XM_017014010.1;NM_001100117.2;XM_017014043.1;XM_017014008.1;XM_017014041.1;XM_017014014.1;XM_017014012.1;XM_017014045.1;XM_017014035.1;NM_014677.4;XM_017014033.1;XM_017014006.1;XM_017014039.1;XM_005251106.3;XM_017014037.1;NM_001282881.1;XM_017014027.1;XM_017014025.1;XM_017014031.1;XM_017014029.1;XM_017014019.1;XM_017014017.1;XM_017014023.1;XM_017014021.1;XM_005251107.3;XM_011517395.2;XM_017014042.1;XM_017014011.1;XM_017014040.1;XM_017014009.1;XM_017014046.1;XM_017014015.1;XM_017014044.1;XM_017014013.1;XM_017014034.1;XM_017014032.1;XM_01	AK308831.1;CR749374.1;CN361794.1;AB018294.1;AK126939.1;BC043144.2	84
Hs01114274_m1	INV	IL24	interleukin 24	C49A;FISP;IL10B;MDA7;MOB5;ST16	XM_011509101.2;NM_006850.3;NM_001185156.1;XM_017000121.1;NM_001185157.1;NM_001185158.1	AY641440.1;BT007156.1;BC009681.1;U16261.1;AY641441.1;AW949784.1;BP222651.1	67
Hs00223273_m1	INV	POPDC2	popeye domain containing 2	POP2	XM_005247698.3;NM_001308333.1;NM_022135.3	AK377182.1;AF204173.1;AK377180.1;AK226022.1;AK377184.1;AK377183.1;AK377174.1;AK377178.1;BC026911.2;AK314819.1;BC044929.1	108
Hs01087516_m1	INV	LRRN3	leucine rich repeat neuronal 3	FIGLER5;NLRR-3;NLRR3	NM_001099660.1	AK001991.1	90
