# Supplementary material for: MYO5B mutations in pheochromocytoma/paraganglioma promote cancer progression
Source: PLoS Genet. 2020 Jun 8;16(6):e1008803. doi: 10.1371/journal.pgen.1008803 (PMC7329139; doi:10.1371/journal.pgen.1008803)
Supplement: S1 File — (PDF) [file pgen.1008803.s012.pdf]

S1 File: Supplementary methods

Variant calling and assessment

Germline variant calling was performed for normal samples (15 N cases) using GATK (v.2.7), according to Wilzén et al [14], and single tumor samples (T) using CLC Genomics workbench (v.4.0, Qiagen) and filtered using Alissa Interpret software (Rev 5.2.2, Agilent Technologies) connected with AlaMut Visual (v.2.11, Interactive Biosoftware/Sophia Genetics). The following filtering criteria for germline variants were applied; read depth >10x, variant allele frequency >=0.20, and population frequency <0.1% (Genome Aggregation Database; GnomAD). Somatic variant calling was performed using paired samples (15 cases) by MuTect (SNVs), Strelka and Varscan 2 (indels) previously described [14]. Somatic variants present in any population database were filtered out. Screening of germline and somatic variants was restricted to a target gene set of 40 previously PPGL-associated genes (S1 Table) [4,7,9,11,12, 13,14,15,18,71,72,73,77]. Only nonsynonymous SNVs or indels in coding regions (exons), splice-sites (+/- 2bp from exon) and -10bp upstream in the promoter region were considered in the variant assessment. Finally, variants predicted "damaging", "deleterious", or "disease causing" by at least 2 out of 3 functional prediction algorithms (SIFT, PolyPhen 2, MutationTaster), and/or variants previously reported in ClinVar (www.ncbi.nlm.nih.gov/clinvar/) or HGMD (portal.biobase-international.com) databases were included in the final variant lists (S2 Table).

Transferrin assay

Staining and localization of Transferrin in stable transfected SK-N-AS cells was visualized (63X) using LSM700 confocal microscope (Carl Zeiss), equipped with Plan-Apochromat 63x/1.40 Oil DIC M27. Confocal settings were as follows: Transferrin (Alexa Fluor 555, Fluorophore #1), DAPI (405, Fluorophore #2): Wavelenght Excited 555 nm (#1) and 405 nm (#2), Emission 573nm (#1) and 435 nm (#2); Laserpower 0.01 (#1) and 0.005 (#2); Offset 11 (#1) and 11.43 (#2); Pinhole diameter 3.42 (#1 and #2); Pinhole size airy 0.78 (#1) and 1.03 (#2); Gain 859.6 (#1) and 919.1 (#2); Zoom 1.5 (#1 and #2).

Expression microarray analysis of SK-N-AS clones

For time point 48h and 72h expression analysis was performed from two different passages of SK-N-AS (p23 and p30, *i.e.* biological replicates), whereas from time point 24h the expression analysis was performed in duplicate from the same RNA (technical replicates) due to failure of automated extraction procedure from one passage. The Clariom S arrays were analyzed using R software v3.4.2. Background correction, normalization and calculation of log2 expression measures for each probe set were performed with the RMA method implemented in the oligo package. Differential expression analysis was performed using the moderated t-statistics implemented in the Limma package, comparing each of the three MYO5B-mutants to $MYO5B^{WT}$ and empty vector, respectively, at three time-points (24h, 48h, and 72h); 18 comparisons in total. To filter the top-candidate genes; the 500 most significant genes (250 up-regulated and 250 down-regulated) with a log2 fold change>0.5 were selected from each comparison. Then, only genes selected both in the comparison to wt and empty vector, respectively, were kept for each mutation, timepoint and direction of regulation (*i.e.* up- or down-regulated). Finally, the resulting lists with top-candidate genes were compared for overlaps between timepoints and mutations. This resulted in a common list of top-ranked differentially expressed genes (overlap results and expression data are presented in S5 Table). The top-ranked genes were were analyzed for enrichment of Gene Ontology terms with GOrilla (Gene Ontology enRIchment anaLysis and visuaLizAtion tool, http://cbl-gorilla.cs.technion.ac.il, [38]), using all genes present at the array as background. In addition, a

Gene Set Enrichment Analysis (GSEA) [39], as implemented in the R-package piano [40] was performed for each mutation and 236 gene sets (50 H Hallmark, and 186 C2: KEGG curated gene sets from the Molecular Signatur database; http://software.broadinstitute.org/gsea/msigdb/index.jsp), using as gene level statistics the mean log2 fold change calculated from 6 comparison per mutant, *i.e.* MUTvsWT and MUTvsEV at three time points.

Microarray expression analysis in primary PCC/PGL

Fresh frozen tumor tissues were homogenized with TissueLyzer and TRIzol reagents (Thermo Fisher Scientific) and total-RNA was extracted using Qiagen RNeasy spin-columns according to standard procedures (Qiagen). All samples had RNA Integrity Number (RIN) between 7-9 using quality assessment by 2100 Bioanalyzer (Agilent Technologies). Microarray analysis was performed by 44K Agilent Cy3/Cy5 2-color microarrays (using Stratagene's Universal Human reference RNA from Agilent Technologies as reference) according to instructions from manufacturer (Version 5.7, March 2008, Agilent Technologies). Analysis was performed in Limma for R 2.9.0 using loess normalization, and a mean expression value (log2) of expressed probe-sets for each target-ID was calculated (probe-sets with log2 expression <0.6 were excluded).
